# Exploring Binding Pockets in the Conformational States of the SARS-CoV-2 Spike Trimers for the Screening of Allosteric Inhibitors Using Molecular Simulations and Ensemble-Based Ligand Docking

**DOI:** 10.3390/ijms25094955

**Published:** 2024-05-01

**Authors:** Grace Gupta, Gennady Verkhivker

**Affiliations:** 1 Keck Center for Science and Engineering, Graduate Program in Computational and Data Sciences, Schmid College of Science and Technology, Chapman University, Orange, CA 92866, USA; grgupta@chapman.edu; 2 Department of Biomedical and Pharmaceutical Sciences, Chapman University School of Pharmacy, Irvine, CA 92618, USA

**Keywords:** SARS-CoV-2 spike protein, ACE2 host receptor, virtual ligand screening, drug design, conformational landscapes, binding energetics, allosteric inhibitors

## Abstract

Understanding mechanisms of allosteric regulation remains elusive for the SARS-CoV-2 spike protein, despite the increasing interest and effort in discovering allosteric inhibitors of the viral activity and interactions with the host receptor ACE2. The challenges of discovering allosteric modulators of the SARS-CoV-2 spike proteins are associated with the diversity of cryptic allosteric sites and complex molecular mechanisms that can be employed by allosteric ligands, including the alteration of the conformational equilibrium of spike protein and preferential stabilization of specific functional states. In the current study, we combine conformational dynamics analysis of distinct forms of the full-length spike protein trimers and machine-learning-based binding pocket detection with the ensemble-based ligand docking and binding free energy analysis to characterize the potential allosteric binding sites and determine structural and energetic determinants of allosteric inhibition for a series of experimentally validated allosteric molecules. The results demonstrate a good agreement between computational and experimental binding affinities, providing support to the predicted binding modes and suggesting key interactions formed by the allosteric ligands to elicit the experimentally observed inhibition. We establish structural and energetic determinants of allosteric binding for the experimentally known allosteric molecules, indicating a potential mechanism of allosteric modulation by targeting the hinges of the inter-protomer movements and blocking conformational changes between the closed and open spike trimer forms. The results of this study demonstrate that combining ensemble-based ligand docking with conformational states of spike protein and rigorous binding energy analysis enables robust characterization of the ligand binding modes, the identification of allosteric binding hotspots, and the prediction of binding affinities for validated allosteric modulators, which is consistent with the experimental data. This study suggested that the conformational adaptability of the protein allosteric sites and the diversity of ligand bound conformations are both in play to enable efficient targeting of allosteric binding sites and interfere with the conformational changes.

## 1. Introduction

The wealth of structural and biochemical investigations conducted on the SARS-CoV-2 virus’s spike (S) glycoprotein has provided crucial insights into the mechanisms that regulate virus transmission and immune evasion. This glycoprotein, tasked with facilitating the virus’s entry into host cells, undergoes significant conformational shifts between closed and open states. These structural transitions are coordinated by the flexible N-terminal S1 subunit, which includes the N-terminal domain (NTD), the receptor-binding domain (RBD), and two structurally conserved subdomains—SD1 and SD2 [1,2,3,4,5,6,7,8,9]. The intricate coordination among these structural domains plays a crucial role in orchestrating conformational shifts within the S protein, transitioning between the closed state with the RBD down and the open state with the RBD up. The efficacy of the S protein in recognizing and binding to host cell receptors, as well as its ability to evade immune detection, relies fundamentally on its capacity to navigate between distinct structural states. Furthermore, the synchronized functional movements of the NTD, RBD, SD1, and SD2 subdomains occur as the S1 subunit undergoes global motions, effectively coordinated through long-range communication with the structurally rigid S2 subunit [10,11,12,13,14,15]. The S1 subunit undergoes conformational transformations between ensembles of closed and open receptor-accessible forms. The S2 subunit is highly conserved fusion machinery that contains an N-terminal hydrophobic fusion peptide (FP), fusion peptide proximal region (FPPR), heptad repeat 1 (HR1), central helix region (CH), connector domain (CD), heptad repeat 2 (HR2), transmembrane domain (TM), and cytoplasmic tail (CT). The S1 domains are situated above the S2 subunit, covering and protecting the fusion apparatus. The essential interactions between the S protein and the host cell receptor ACE2, as well as the diverse range of interactions with different classes of antibodies, are significantly influenced by the collaborative interplay of functional motions involving both S1 and S2 subunits. This interplay plays a pivotal role in mediating these interactions and governing the broad spectrum of S interactions, consequently impacting the host immune responses triggered by the virus. 

The extensive insights gained from biophysical studies have improved our comprehension of the S protein trimer, illuminating the intricate interplay between thermodynamics and kinetics that govern spike mechanisms. These investigations have revealed a nuanced understanding of how mutations and long-range interactions between the dynamic S1 subunit and the more rigid S2 subunit orchestrate coordinated structural alterations within the S protein trimer. Recent experimental studies suggested that dynamic biological functions of the SARS-CoV-2 S proteins and mutational escape mechanisms can be rationalized and predicted by examining critical molecular events related to viral infection and dissemination through the lens of allosteric regulatory landscape of the SARS-CoV-2 S protein [16,17,18]. Conformational dynamics of the SARS-CoV-2 S protein in the absence or presence of ligands visualized using smFRET imaging assays showed that ACE2 binding is controlled by the conformational landscape of the RBD via population-shift mechanism, in which ACE2 captures the intrinsically accessible up RBD conformation rather than inducing a conformational change [18]. Moreover, smFRET data demonstrated that antibodies that target diverse epitopes of the S protein located away from the RBD can allosterically modulate the RBD functional dynamics and shift the thermodynamic equilibrium towards the open S form that promotes ACE2 binding. Conformational dynamics of the SARS-CoV-2 trimeric S glycoprotein in complex with ACE2 revealed by cryo-EM experiments further confirmed that binding can modulate conformational landscape of the S trimer [19]. The cryo-EM and crystal structures of the S protein and complexes with ACE2 and antibodies provided a significant wealth of accurate high-resolution structural information and unveiled diversity of conformational states sampled by Omicron variants. Nonetheless, the details of the conformational dynamics are often hidden or obscured due to the rapid interconversion of states and the complexity of the binding-induced modulation of flexibility seen in the experiments. 

Hydrogen/deuterium exchange mass spectrometry (HDX-MS) is a powerful approach for monitoring local protein structural dynamics under native solution conditions; by tracking deuterium uptake kinetics for peptide segments throughout a given protein, this approach can reveal dynamics and residue-specific changes in the conformational dynamics induced by mutations or binding. HDX-MS studies uncovered an alternative open S trimer conformation that interconverts slowly with the known prefusion structures and can dynamically expose novel epitopes in the conserved region of the S2 trimer interface, unexpectedly suggesting the emergence of hidden cryptic epitopes in a highly conserved region of the protein [20]. Another HDX-MS study identified changes in the S dynamics for variants of concern (VOCs), revealing that Omicron mutations may preferentially induce closed conformations with dynamic core helices in the S2 subunit exploited in the fusion stage, and that the NTD acts as a hotspot of conformational divergence, driving immune evasion [21]. Comparative HDX-MS analysis of the WT, D614G, Alpha, Delta, and Omicron S variants showed that Omicron mutations can induce greater stabilization of the trimeric stalk interface in the S2 subunit concurrently with the increased NTD and RBD dynamics which may have direct implications for ACE2 binding [22]. HDX-MS studies of the unbound S protein trimer also revealed the highest relative exchange in the S2 subunit and the stalk regions (central helix CH and heptad repeats HR1 and HR2), while a comparative analysis of the S protein and S–ACE2 complex suggested that ACE2 binding allosterically enhances dynamics at a distal S1/S2 cleavage site and stalk hinge region [23]. Another HDX-MS study of S–ACE2 binding confirmed that the ACE2 can induce enhanced dynamics throughout the entire S protein, with allosteric changes being propagated to the S2 hinge region and to the central helical bundle of the S2 subunit [24]. 

The cryo-EM structure of the S protein with linoleic acid (LA) complex revealed allosteric binding pocket in the S-RBD that is controlled through opening of a gating helix and can allosterically induce stabilization of the closed S trimer and promote decreased S–ACE2 binding [25,26,27,28]. Notably, in the experimental S trimer, complex LA binding occurs in a bipartite binding pocket in which the hydrophobic tail of the LA molecule is bound to the RBD on one protomer, and the carboxyl headgroup of the LA interacts with the R408 and Q409 of the neighboring RBD [26,27]. These studies underscored the importance of identifying conserved cryptic epitopes as essential targets for the design of universal vaccines and broadly neutralizing antibodies [29]. The recent structural investigations unveiled another cryptic binding pocket located in the NTD region, also demonstrating that tetrapyrrole products of heme metabolism, biliverdin and bilirubin, can bind to this site with nanomolar affinity and inhibit neutralizing activity of the NTD antibodies targeting these epitopes [30,31,32]. Through integration of computational and experimental methods, a new cryptic allosteric site located between subdomains of the S protein was discovered and experimentally validated, with several compounds targeting this site showing micromolar binding and anti-virus activities [33,34,35]. These studies discovered an allosteric binding pocket at the junction of the S1 and S2 subunits, which does not overlap with the RBD showing that binding of this region could allosterically inhibit spike function by stabilizing the closed state of the spike RBD [33]. By screening commercial compound databases, several compounds with high binding affinity for this region (denoted CPD7, CPD20, and CPD26) were discovered and implicated in targeting two main areas inside the binding pocket [33]. Several of the CPD compounds, especially CPD26 and CPD7, showed strong inhibitory effect, suggesting that these compounds may bind to the proposed cryptic inter-protomer pocket and allosterically inhibit viral entry. Using a combination of computational methods and biochemical tools, N-Acetylneuraminic acid (Neu5Ac), a type of predominant sialic acid found in human cells, was evaluated as a molecular probe of the S protein and validated as an allosteric modulator [35]. A strategy combining molecular docking and surface plasmon resonance (SPR) analysis of compound libraries interrogated 57,641 compounds and identified 17 binders of ACE2 and 6 potent blockers of the RBD that compete with the RBD–ACE2 interactions [36]. Structure-based virtual screening against the LA-binding allosteric pocket on the RBD identified a panel of allosteric modulators with several hits displaying micromolar binding affinities that were confirmed by the cryo-EM structure of S protein bound with the allosteric modulators [37]. 

Large scale simulations of the viral proteome captured conformational heterogeneity of the S protein and predicted the existence of multiple cryptic epitopes and hidden allosteric pockets [38]. The replica exchange molecular dynamics (MD) simulations examined conformational landscapes of the full-length S protein trimers, discovering the transition pathways via inter-domain interactions, hidden functional intermediates along open-closed transition pathways, and previously unknown cryptic pockets that were consistent with FRET experiments [39]. The cryo-EM metainference (EMMI) method modeled conformational ensembles by combining simulations with cryo-EM data, revealing the intermediate states in the opening pathway of the S protein and discovering potentially druggable novel cryptic sites near the RBD recognition site [40]. Our recent studies combined multiscale simulations with network-based modeling to reveal that the S protein can function as a functionally adaptable allosterically regulated machine that exploits the plasticity of allosteric centers to fine-tune responses to antibody binding [41,42,43,44,45]. Recent computational studies in our laboratory also showed that SARS-CoV-2 S Omicron mutations have variant-specific effects on conformational dynamics changes including allosterically induced plasticity at the remote regions leading to the formation and evolution of druggable cryptic pockets [46,47]. 

Computer simulation-based mapping of the full-length models of the S protein in the presence of benzene probes reproduced experimentally discovered allosteric sites and identified a spectrum of novel cryptic and potentially druggable pockets [48]. MD simulations of the unbound or ACE2-bound RBD conformations combined with pocket analysis and druggability prediction identified several promising druggable sites, including one located between the RBD monomers [49]. Recent functional studies identified that the metabolite of fenofibrate (fenofibric acid) destabilized the RBD protein and significantly reduced infection rates in vitro by inhibiting the ACE2 binding [50]. MD simulations and energetic analysis suggested that fenofibric acid induces a conformational change in the RBD by stabilizing a potential cryptic binding site on the RBD [51]. Another study detected a potential allosteric pocket within the S protein and conducted large-scale structure-based virtual screening and MD simulations by targeting the putative allosteric pocket to identify allosteric inhibitors and evaluate the effects of compound binding on the stability of the RBD [52]. High-throughput screening of 30,000 small molecules was conducted with the full-length S protein, showing that two compounds, oleic acid and suramin, can block binding with ACE2 using a noncompetitive mechanism [53]. Our recent study examined allosteric binding sites in the S protein and binding mechanisms of free and bound forms of spike with hepcidin (HPC), the major hormone implicated in the COVID-19 pathogenesis, showing the possibilities of the allosteric inhibition mechanism [54]. A combined proteomics and computational investigation discovered two thiol-based chemical probes, P2119/P2165, that decrease the S binding to ACE2 by disrupting the Cys379–Cys432 and Cys391–Cys525 pairs in the RBD core and inducing conformational changes that are consistent with an allosteric mechanism of inhibition [55]. Utilizing screening data from the SARS-CoV-2 cytopathic effect reduction assay, two iterative rounds of virtual screening of small molecules were then performed against SARS-CoV-2, resulting in the identification of ACE2 allosteric inhibitors [56]. A large-scale computational study presented a multiple-step in silico screening protocol to identify allosteric inhibitors of S–ACE2 binding in which a compound library containing 77,448 antiviral compounds was screened using molecular docking and binding affinity calculations [57]. The use of screening of library of organic dyes and related drug-like compounds led to the identification of several small-molecule compounds, showing promising broad-spectrum inhibition of the S–ACE2 interactions acting as blockers of viral attachment for SARS-CoV-2 [58].

Despite this progress, the identification and characterization of small molecules inhibiting S–ACE2 interactions and particularly allosteric modulators continues to be quite challenging due to the conformational heterogeneity of the S protein, diversity of multiple cryptic binding sites, and elusive nature of allosteric inhibition that is often difficult to evaluate due to long-range effects and effects on the conformational equilibrium. Only several small molecules such as clofazimine that potentially inhibit SARS-CoV-2 spikes have reached late-stage clinical trials. Some reported small-molecule inhibitors of the S–ACE2 interaction such as MU-UNMC-2, P2119, P2165, H69C2, DRI-C23041, and AB-00011778 undergo optimization before they could progress towards clinical trials [59]. To counteract resistance, it is important to develop potent agents and their combinations that target conserved binding pockets within or outside the RBD. In addition, it is important to quantify structural and energetic aspects of the observed activity of small molecules as they often do not act on the presumed pharmacological targets, but rather interfere with viral replication through non-specific effects and can therefore exploit off-target mechanisms based on their physicochemical characteristics.

In the current study, we employed an integrative approach that included detection and characterization of allosteric pockets in the S-RBD and S trimer proteins, coarse-grained Brownian dynamics (CG-BD) simulations with atomistic reconstruction of equilibrium trajectories of the S protein timers, ensemble-based ligand docking against conformational ensembles of the S closed trimers, and binding free energy evaluation for allosteric modulators. Using ensemble docking screening of small molecules against several allosteric binding pockets of the S protein, we examined structural and binding preferences of ligand libraries against major allosteric binding sites in the full-length S protein trimer that were experimentally validated as targets of allosteric inhibitors. These binding sites included the allosteric site located at the junction of SD1 and SD2 and flanked by residues F541, K557, and I587 on one protomer and I834, F855, M740, and D745 of the neighboring protomer (referred to as site A) [33] and the allosteric site targeted by sialic acid (referred to as site B) which is flanked by residues R403, D495, R408, S375, and K378 [35]. The first set of docked small molecules (referred to as Library 1) consisted of 200 drug-like compounds that showed in vitro activity against SARS-CoV-2 [57]. This library of drug-like molecules was screened to (a) evaluate whether the two experimentally validated allosteric binding sites in the S trimers could serve as a drug binding pocket for these compounds and (b) determine structural and energetic determinants of ligand binding in the known allosteric binding sites and suggest putative allosteric inhibitors based on the rigorously computed binding energetics. We identified several small molecules targeting both allosteric pockets, interacting with the allosteric hotspots and displaying high binding affinity against both allosteric sites. A close chemical similarity between the top predicted hits and experimentally validated compounds inhibiting the S protein attachment to ACE2 receptor suggested the potential for the allosteric mode of inhibition. 

We also examined a library of the experimentally validated allosteric molecules denoted CPD1–CPD26 in the original study [33]. In particular, three discovered allosteric modulators against SARS-CoV-2, CPD7, CPD20, and CPD26, were confirmed to exhibit the allosteric mechanism and suggested to act by indirect binding outside of the RBD and inhibiting conformational transitions of the S trimer [33]. However, the atomistic details of structural and energetic determinants of allosteric inhibition remain unknown. We simulated binding of this library of allosteric modulators to the two allosteric sites and characterized structural and binding mechanisms for these molecules. We also identified key interactions and allosteric hotspot residues in the S protein targeted by the allosteric compounds and compared predicted binding affinities of these molecules with the experimental SPR-measured binding constants [33]. By complementing structural predictions with rigorous binding affinity computations, we showed good agreement between computational and experimental binding constants for this library of allosteric molecules. The results of this study reveal structural and energetic determinants of allosteric inhibition for the experimentally known allosteric molecules, pointing to a mechanism of allosteric modulation by targeting the hinges of the inter-protomer movements in site A and blocking conformational changes between the closed and open S trimer forms. This study may have implications for understanding the effects of protein heterogeneity and an ensemble-based view of ligand binding, providing useful insights into mechanisms of the allosteric inhibition of S proteins.

## 2. Results and Discussion

### 2.1. Identification and Characterization of Cryptic Pockets and Allosteric Binding Sites in the S Trimers: Hinge Positions of Functional Interprotomer Motions Form the Targeted Site for Allosteric Modulators

**Figure 1:**
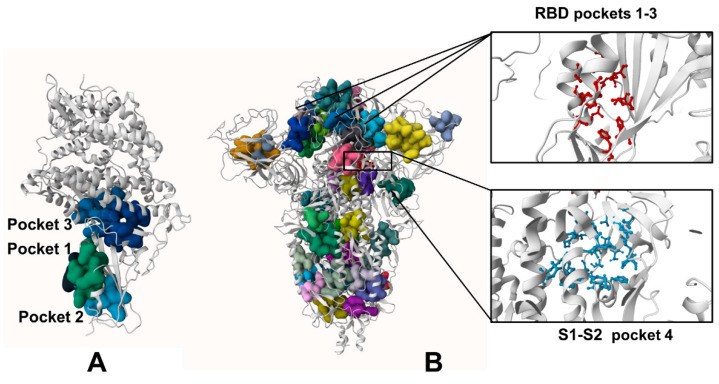
The predicted binding pockets in the S-RBD structure and the full-length S protein trimers. (**A**) The top three predicted pockets for the S-RBD: pocket 1 (B_365, B_368, B_369, B_377, B_387, B_432, B_434, B_513, B_515); pocket 2 (B_343, B_344, B_345, B_347, B_371, B_372, B_436, B_441, B_509), third ranked pocket 3 (A_321, A_324, A_350, A_353, A_354, A_356, A_383, A_386, A_387, A_393, B_405, B_503, B_504, B_505). The top ranked RBD pocket 1 corresponds to the known LA allosteric binding site. (**B**) Structural map and residue-based close-ups of the top ranked predicted binding sites for the ensemble of the S closed trimers. Pockets 1, 2, and 3 correspond to the LA allosteric pockets on protomers A, B, and C, respectively. Pocket 4 corresponds to the inter-domain allosteric binding site determined in the experimental screening of allosteric inhibitors [33]. The complete list of the S residues that constitute the predicted binding pockets are presented in Supporting Information, Table S1. The notations A_number, B_number, and C-number annotate the S protein residue numbers of the protomers A, B, and C, respectively. For example, A_570 and B_365 annotations refer to residue 570 on the protomer A and residue 365 on the protomer B.

We performed a detailed quantitative analysis of the top ranked pockets in the S-BA.1 and S-BA.2 ensembles to connect computational predictions guided by the allosteric role of the binding residues with the existing experimental data and functional knowledge of S protein mechanisms. We used two different complementary approaches for the identification of the cryptic binding pockets, P2Rank [60] and PASSer (prediction of allosteric sites server) [61,62,63]. To understand the predictive ability of the approach and decipher the functional role of the cryptic pockets, we started by comparing complete structural maps of all identified pockets versus the top ranked cryptic sites for the S-RBD and the full-length S trimer. This analysis revealed important similarities and differences underlying the emergence of allosteric binding pockets in the S protein (Figure 1). First, we predicted cryptic pockets in the S-RBD complex with ACE2 using the structure of Wu-Hu-1 RBD-ACE2 (pdb id 6M0J). The pocket detection analysis revealed three highly probable pockets (Figure 1A). The first RBD pocket is lined up by residues Y365, S366, Y369, F377, V382, S383, P384, L387, N388, F392, C432, and F515, and the second RBD pocket 2 is formed by residues F338, V341, F342, N343, A344, V367, L368, N370, A372, F374, W436, N437, L441, and R509 (Figure 1A). The residues that form these pockets, namely a major group of hydrophobic allosteric centers from the RBD core (F338, V341 F342, and W436), form a well-defined conserved allosteric binding pocket, and this pocket corresponds precisely to the experimentally determined allosteric site where the essential free fatty acid LA binds [26.27]. LA occupies a site formed between two adjacent RBDs, and considering that the S protein is a trimer, three similar pockets are defined, with up to three molecules of LA capable of binding simultaneously. This cryptic allosteric pocket is highly probable and conserved even in the absence of the adjacent protomers. Interestingly, the third ranked pocket 3 (A_321, A_324, A_350, A_353, A_354, A_356, A_383, A_386, A_387, A_393, B_405, B_503, B_504, and B_505) is formed at the RBD–ACE2 interface and includes RBD residues D405, V503, G504, and Y505 (Figure 1A, pocket 3). This pocket includes residues that were implicated in the binding site for sialic acid molecules (referred to as site B in this study) which symmetrically bind around the residue of D405 in each protomer with forming couples of molecular interactions, such as the hydrogen bonds with G504, G404, and K417 [35]. Hence, the molecules targeting this site may function as inhibitors of the S activity by interacting with the interfacial pocket and binding between the two RBDs of the S protein, thus disrupting the RBD–ACE2 binding interactions rather than allosterically altering RBD conformation. 

The detection of potential binding pockets in the S-RBD provides only a fraction of the pockets that are available in the S protein trimer structure (Figure 1B). The detected binding pockets in the closed S protein trimer form a dense network of overlapping sites which may be relevant for propagating signals in the S protein. However, only a relatively small fraction of the S1 binding pockets have functional significance for allosteric conformational changes and could potentially serve as target binding sites for allosteric inhibitors. Allosteric inhibitors can modulate protein activity in several ways namely by affecting conformational changes and functional motions between the closed and open S forms or by disrupting protein-protein interactions. Given the experimentally known allosteric sites and probable mechanisms underlying allosteric inhibition, we particularly focused on the analysis of binding pockets that are anchored by functionally important for dynamics residues or reside at the inter-protomer and S1–S2 interdomain interfaces. To address this conjecture, we focused on the closed forms of the S protein trimer, as these functional states can be the well-defined architecture of the binding pockets and particularly feature well-shaped conserved inter-protomer pockets that could serve as allosteric binding sites where ligand targeting could “freeze” conformational equilibrium and prevent the S protein trimer from adopting an open form required for efficient binding with the ACE host receptor.

The top ranked LA pocket in the S closed trimer is formed by RBD residues F338, I358, A363, Y365, L368, Y369, F377, L387, F392, V395, C432, I434, L513, and F515 on each protomer (Figure 1B, pockets 1–3 for protomers A, C, and B). This prediction closely matched the experimentally known LA allosteric site that is anchored by several phenylalanine residues (F338, F342, F374, F377, F392, and F515), and some other hydrophobic amino acids (C336, P337, I358, A363, Y365, L368, Y369, L87, V395, I434, L513, and V524) [26,27,28]. At the entrance of the pocket, the adjacent RBD from the other protomer exposes several hydrophilic residues, R408, Q409, G416, and K417, that contribute to the pocket by forming both electrostatic and hydrogen-bond interactions [26,27,28]. Potential allosteric ligands would interact with residues in both portions, and the presence of the adjacent RBD is required to stabilize the ligand by forming direct interactions with the hydrophilic groups. Hence, computational prediction can predict the experimentally known allosteric site as the top ranked binding pocket (Figure 1). There are several experimentally validated allosteric inhibitors that bind to the LA binding pocket and exert a biological activity by allosterically inhibiting the S–ACE2 interaction, as was determined by SPR and cryo-EM analyses [36.37]. Several studies described other potential allosteric ligands with a confirmed binding in vitro assays showing a good occupation of the LA pocket, indicating that compounds binding to this allosteric site with high affinity have the potential to stabilize the inactive conformation of the S protein [64]. 

The pocket analysis predicted the experimentally known LA allosteric pocket [26,27,28] as the top ranked pocket in the S trimer and this pocket is formed on each of the respective protomers A, B, and C. The next top ranked pocket in the S trimer is the inter-protomer site formed near the S1–S2 hinge region that bring S1 residues on one protomer (positions 541, 546, 547, 548, 549, 570, 572, 573, 587, 589, and 592) and S2 residues from the adjacent protomer (positions 1000, 740, 741, 744, 745, 855, 856, 966, 976, 977, and 978) (Figure 1B, pocket 4) [33] This allosteric site is located at the junction of SD1 and SD2 and flanked by residues F541, K557, and I587 on one protomer and I834, F855, M740, and D745 of the neighboring protomer. Importantly, this predicted pocket was experimentally validated as an allosteric site and serves as a binding site for a series of potent allosteric modulators [33]. We refer to this binding pocket as site A that will be used for molecular docking and screening of small molecules and allosteric modulators. Our results suggested that the predicted top binding pockets in the S trimer may be possible candidates for allosteric modulators affecting conformational dynamics and structural transitions between functional closed and open states. 

### 2.2. Simulations of the SARS-CoV-2 Spike Trimers and Analysis of Functional Motions Identify Inter-Domain Hinges Controlling Conformational Equilibrium between Open and Closed States

**Figure 2:**
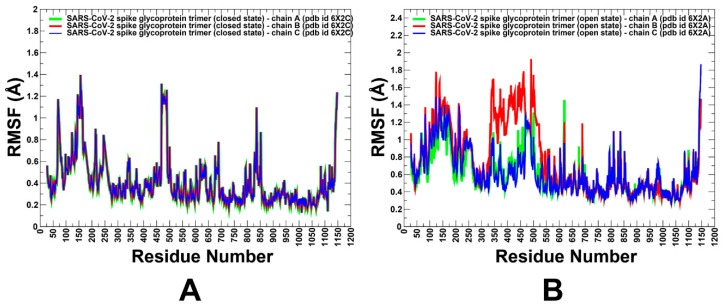
Conformational dynamics profiles of the SARS-CoV-2 spike (S) protein trimer in the closed (**A**) and open states (**B**) of the prefusion form. (**A**) The RMSF profiles from simulations of the cryo-EM structure of SARS-CoV-2 S trimer in the closed state (pdb id 6X2C). (**B**) The RMSF profiles from simulations of the cryo-EM structure SARS-CoV-2 S trimer ectodomain in the open state (pdb id 6X2A). The profiles for protomer chains A, B, and C are shown in green, red, and blue lines, respectively.

First, to characterize and quantify structural and dynamic drivers of allosteric changes in the S trimers, we performed coarse-grained molecular simulations followed by full atomistic reconstruction of the equilibrium trajectories and functional dynamics analysis of the S trimer proteins in the closed and open forms (Figure 2). MD simulations of the full-length SARS-CoV-2 S structures in different functional states with a complete glycosylation shield profile and rigorous solvent description are incredibly time-consuming and require enormous resources to execute for multiple SARS-CoV-2 S structures in fully glycosylated environments. Using CG-BD simulation approaches applied to full atomistic high-resolution structures of the SARS-CoV-2 S prefusion trimer in multiple functional states, we performed a detailed comparative analysis of the conformational dynamics profiles and examined salient features of the dynamic conformational landscapes for the SARS-CoV-2 S trimer (Figure 2). Simulations of the closed and open forms of the perfusion state revealed important dynamic signatures of these states (Figure 2). A comparative analysis of the dynamics’ profiles showed generally very moderate thermal fluctuations of both the S1 and S2 subunits in the closed state (Figure 2A). While the NTD regions and the furin cleavage site (residues 680–688) showed larger displacements, the mobility of the RBD residues was markedly suppressed in this form compared to more dynamic closed and open prefusion states (Figure 2).

We observed that both closed and open states featured significantly larger thermal fluctuations in the NTD and RBD regions, particularly of the exposed RBM segment involved in recognition of the host receptor. The root mean square fluctuations (RMSF) profiles obtained from simulations showed an appreciable and consistent difference between a considerably more flexible S1 subunit and generally structurally rigid S2 subunit in all functional states (Figure 2). The NTD residues 14–306 and RBD residues 331–528 of S1 showed flexibility in both forms. CTD1 (residues 529–591) is a structural relay between the RBD and S2 regions that can arguably sense the functional movements in the S1 and S2 domains and communicate signals from and to the fusion peptide. These segments of the S1 subunit are largely stable in all functional states of the S trimer (Figure 2). The S2 subunit exhibited small thermal fluctuations with RMSF < 1.0 Å, which is consistent with strong sequence conservation in these regions. The following small thermal fluctuations are seen for the S2 regions: upstream helix (UH) (residues 736–781), heptad repeat 1 (HR1) (residues 910–985), and central helix (CH) (residues 986–1035) (Figure 2). As expected, CG-BD simulations of the open form of the trimer revealed the large fluctuations in the “up” monomer that becomes exposed for interactions with the host receptor (Figure 2B). Domains HR1, CH, and CD close to the viral transmembrane exhibited the least movement during simulations. In general, the results showed that both closed and open forms of the S trimer can maintain flexibility of the S1 subunit required for dynamic transformations of RBDs. We particularly focused on analyzing dynamics of the S residues near allosteric sites A and B. Allosteric site A is located at the junction of SD1 and SD2 and flanked by residues F541, K557, and I587 on one protomer and I834, F855, M740, and D745 of the neighboring protomer. The dynamics profiles showed conformational adaptability at the S1–S2 junction and greater flexibility due to local changes around hinge sites. At the same time, the allosteric site B, which is flanked by residues R403, D495, R408, S375, and K378, displayed smaller fluctuations. The greater stability of site B is associated with stability of the RBD–RBD interfaces in the closed trimer. However, some level of conformational plasticity is present in site B, albeit it is more stable than site A. Our results suggested that conformational variations in site A may be associated with the inter-domain adjustments that also control transitions between the closed and open states.

**Figure 3:**
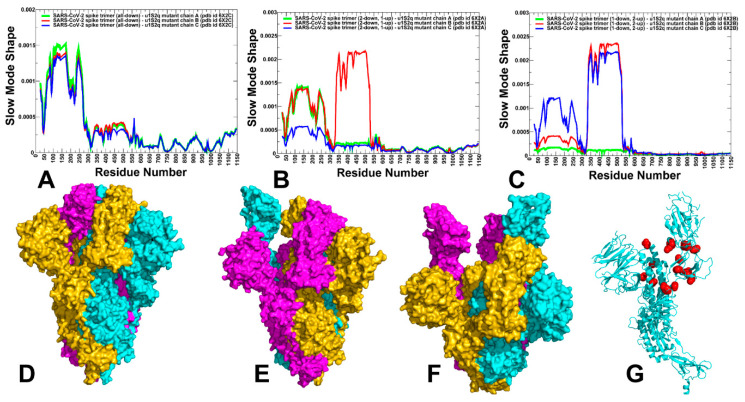
The slow mode mobility profiles of the SARS-CoV-2 S trimer structures. The slow mode dynamics profiles represent the displacements along slow mode eigenvectors and correspond to the of the slowest 10 modes. (**A**) The slow mode mobility profiles for the closed S closed trimer structure (pdb id 6X2C). (**B**) The slow mode mobility profiles for the 1RBD-up open S trimer structure (pdb id 6X2A). (**C**) The slow mode mobility profiles for the 2RBD-up open S trimer structure (pdb id 6X2B). The slow mode profiles for protomer chains A, B, and C are shown in green, red, and blue lines, respectively. (**D**) The closed S closed trimer structure (pdb id 6X2C). (**E**) The 1RBD-up open S trimer structure (pdb id 6X2A). (**F**) The 2RBD-up open S trimer structure (pdb id 6X2B). The S protein structures are shown in surface with protomers A, B, and C in green, light pink, and magenta colors, respectively. The local minima along slow mode profiles correspond to hinge sites involved in collective movements of the S trimer. (**G**) Structural map of the hinge positions (shown in red spheres) onto a single protomer of the closed S trimer (light pink ribbons).

To characterize collective motions and determine the distribution of hinge regions in the SARS-CoV-2 S trimers, we performed PCA of trajectories (Figure 3). The slowest modes and reported functional dynamics profiles were averaged over the first 10 major low frequency modes. The conserved hinge regions that can regulate the inter-domain movements between RBD and NTD are localized at F518 and F541 positions (Figure 3). These hinges are responsible for coordination of functional movements of the NTD and RBD with respect to S2 subunit and are shared by all states. The hinge positions correspond to residues S383, K386, F541, T547, L570, I572, S591, F592, S750, Y855, I856, T962, and D985 where F592 position in one protomer can form the inter-protomer cluster with K854, F855, N856, and T859 hinge sites of the adjacent protomer. Importantly, F541, L570, I572, Y855, I856, and S591 hinge resides are situated near the inter-protomer and inter-domain SD1-S2 interfaces that function as regulatory switch centers governing the population shifts between closed and open forms. Hence, site A which is formed at the inter-domain juncture is enriched by regulatory positions critical for functional S1–S2 movements and transitions between the open and closed S states. As a result, high affinity molecules binding to this pocket may affect conformational changes between functional states and serve as allosteric modulators of the S activity. To summarize, our analysis showed that site A is an allosteric site as targeting this pocket can allosterically alter the conformation of the S protein. At the same time, molecules targeting site B may function by disrupting the RBD–RBD binding interactions rather than exploiting hinge-based allosteric mechanisms. 

In the following sections, we probe these allosteric binding sites using ligand docking to characterize structural and energetic aspects of binding to these sites. We also utilize the experimentally validated allosteric molecules to probe their binding to the two allosteric sites and compute binding affinities to compare computational and experimental binding constants. Through comparison with the experimental data, we analyze mechanisms by which these allosteric molecules can inhibit the S protein activity. 

### 2.3. Ensemble-Based Molecular Docking of Drug-like Molecules to Distinct Binding Sites of the S Trimers: Exploiting Conformational Landscapes of the S Protein for Quantifying Dynamic Binding Preferences of Small Molecules 

The ensemble-based molecular docking screened molecules using the structure and equilibrium conformations of the full-length S trimer. The objective of this molecular screening was three-fold: (a) to examine the structural and energetic binding profiles for library of drug-like molecules with in vitro activity against S protein using the full-length S trimer structures; (b) to probe a potential allosteric hotspots and identify key binding interactions for allosteric inhibition scenarios by targeting the inter-protomer S1–S2 allosteric site A and targeting the RBD–RBD interactions at site B; (c) to probe the experimentally validated allosteric pockets with a panel of allosteric modulators and through comparison of computational predictions with the experimental binding constants characterize potential mechanism of inhibition. 

**Figure 4:**
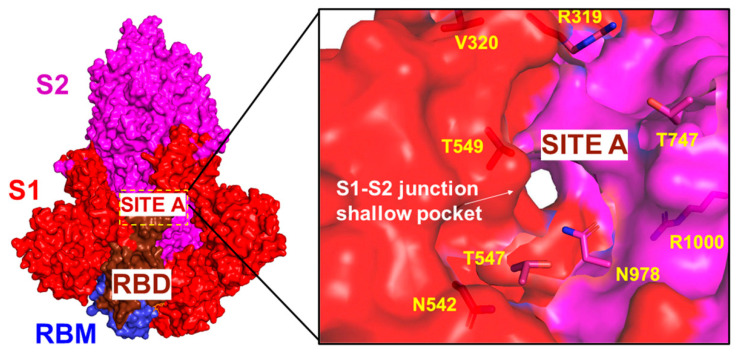
A closeup of the inter-protomer site A [33]. Site A located at the junction of SD1 and SD2 and flanked by residues F541, K557, and I587 on one protomer and I834, F855, M740, and D745 of neighboring protomer (referred as site A) [33]. The cryo-EM structure of the closed S trimer (pdb id 6VXX, 6X2C) is used. The S1 subunit in red surface and S2 subunit in magenta surface. The key residues forming the inter-protomer pocket are annotated and shown in red sticks for S1 residues and magenta sticks for S2 residues.

We performed ensemble-based molecular docking to the binding pockets with the focus on the allosteric site A (Figure 4A) and allosteric site B (Figure 4B) by using simulations of the S closed trimer (pdb id 6VXX, 6X2C) (Supporting Information, Tables S2–S4). 

**Figure 5:**
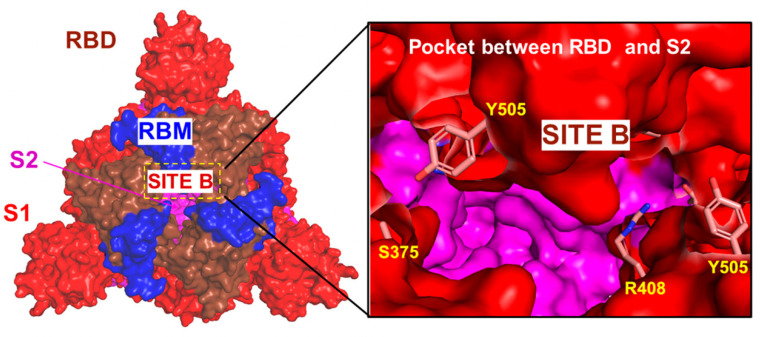
A closeup of site B formed by residues R403, D495, R408, S375, and K378 [35] Site B is centered on residues Y505, R408, and K378 of the S1 subunit. The cryo-EM structure of the closed S trimer (pdb id 6VXX, 6X2C) is used. The S1 subunit in red surface and S2 subunit in magenta surface. The S1 residues are shown in sticks and annotated.

The ensemble of protein conformations was extracted from equilibrium trajectories by taking 100 snapshots from the trajectory distributed equally in time. This approach provided a comprehensive analysis of ligand binding modes and protein mobility during docking. A library of drug-like molecules and a smaller library of validated allosteric modulators were compiled for screening against site A (Figure 4) and site B (Figure 5). Library 1 consisted of 200 drug-like compounds from ZINC database that showed SARS-CoV-2 in vitro activity [57]. For this library, we used the compounds with the highest affinity for the two druggable sites on the RBD [57]. The top 200 compounds from this collection were employed for ensemble-based screening against allosteric binding sites in the S trimer. The central objective of this ligand screening was to characterize structural and energetic basis of ligand binding to the different allosteric pockets, identify preferential allosteric site for ligands displaying strong binding affinity, and suggest potential allosteric inhibitors based on the rigorously computed binding energetics. Docking was performed using AutoDock Vina [65] for the multiple conformations of the S trimer extracted from equilibrium trajectories. The ensemble-based docking calculation consisted of 200 independent runs against 100 equilibrium protein conformations. Each independent run was started with a ligand in random conformation, orientation, and torsions inside the docking grid box [65]. 

**Figure 6:**
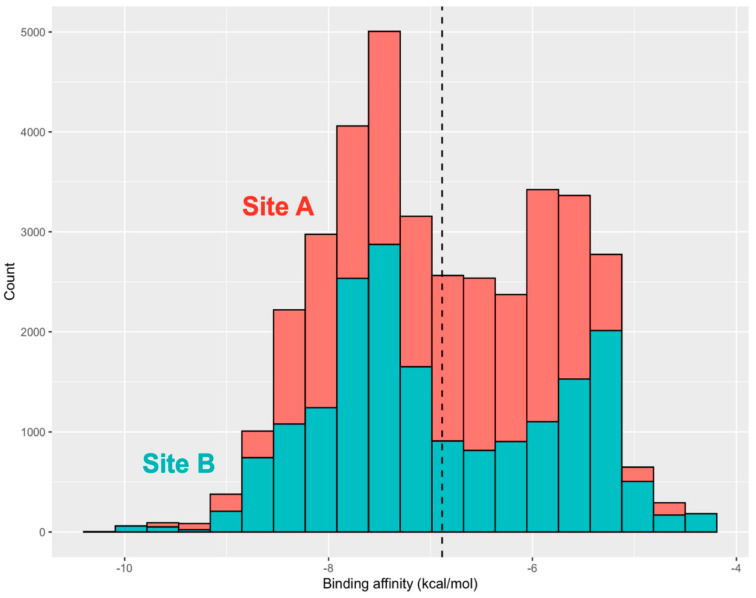
Comparison of binding affinity distributions for all 200 compounds in Library 1 (all 200 iterations included) evaluated against site A and site B. The histogram of binding scores for site A is shown in red bars and for site B in blue bars.

We begin by analyzing the distribution of binding affinity for all iterations of the 200 compounds in Library 1 at site A and site B (Figure 6). Library 1 had an average binding affinity of −7 kcal/mol across all tested compounds against both sites. The distribution of site B was slightly skewed towards higher binding affinity compared to the distribution of site A. The interesting feature of this distribution is that the average binding energetics of bound complexes for this library of drug-like molecules is more favorable at the allosteric site A and continues to be preferable for the lowest energy tail of the distribution (Figure 6). Notably, however, some of the very lowest binding energies were detected for ligands bound in site B, but the frequency and population of binding poses at site B is considerably lower.

**Figure 7:**
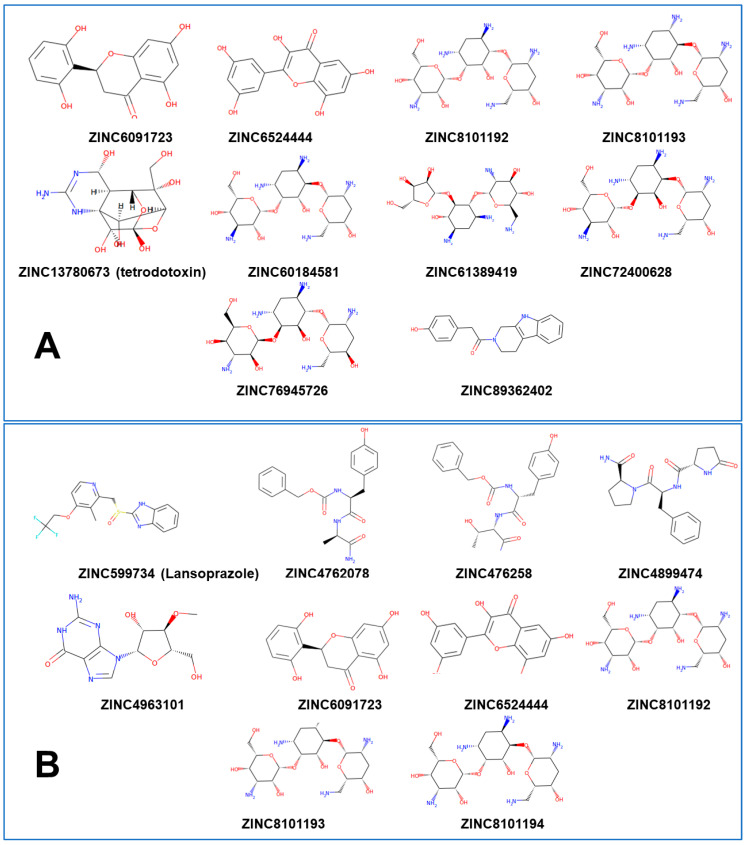
(**A**) The chemical formula and ZINC numbers [66,67,68] of the top 10 molecules based on their median binding energy at site A. (**B**) The chemical formula and ZINC numbers [66,67,68] of the top 10 molecules based on their median binding energy at site B.

The high binding affinity scores of the top lead compounds are −9.7 kcal/mol for site A and −9.0 kcal/mol for site B (Figure 6). Interestingly, the median binding affinity of the lead compounds was similar for site A and site B, ranging between −8 kcal/mol and −9 kcal/mol and suggesting that the best binding molecules may potentially target either one of these binding pockets (Figure 6). At the next step, the top 10 lead compounds for each of the sites were identified and selected based on their median binding affinity (Figure 7). Interestingly, we found that among the top 10 compounds for each site, some molecules could target both sites and have high binding scoring, while there were also site-specific high scoring compounds (Figure 7).

**Figure 8:**
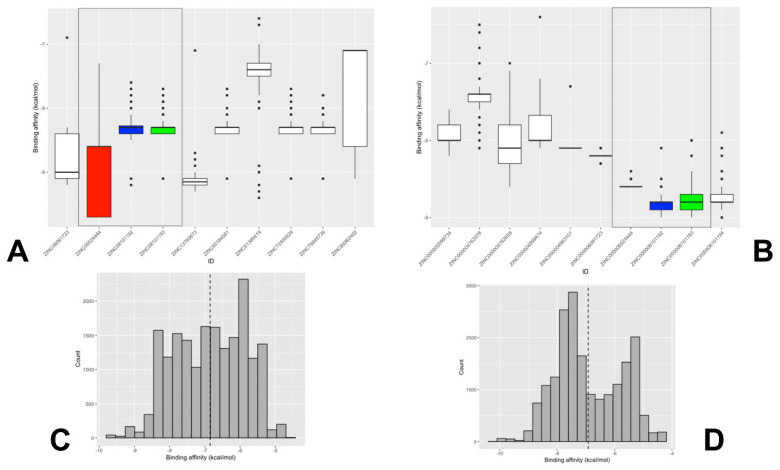
The AutoDock binding affinity distributions for the top 10 screened compounds at site A and site B. The box and whisker plot of binding affinity distributions for the top 10 screened compounds at site A (**A**) and site B (**B**) highlights the distribution of data points. The left and right sides of each box are the lower and upper quartiles. The box covers the interquartile interval, where 50% of the data are found. The vertical line that splits the box in two is the median. The data points correspond to 200 docking scores obtained for each compound from 200 independent ensemble docking runs. The box and whisker plots are highlighted for the three top scoring compounds that can target both sites: ZINC06524444 (in red), ZINC08101192 (in blue), and ZINC08101193 (in green). (**C**) The binding affinity histogram distributions for the top 10 screened compounds at site A (**D**) The binding affinity histogram distributions for the top 10 screened compounds at site B.

The ensemble-based docking to site A resulted in an appreciable dispersion of binding energies and a multitude of potential binding poses (Figure 8A). This may be explained by protein variations around the SD1–SD2 junction and local movements of residues in this region. As a result, although the shape and composition of site A remain intact, local conformational changes can induce the increased variability of ligand binding poses in this region. Given the smaller size of site B, lead compounds from site B displayed decreased variance in binding affinity compared to those at site A (Figure 8B). We also examined the energy histogram distribution profiles for the best scoring molecules from library 1 in site A (Figure 8C) and site B (Figure 8D). Interestingly, the energetic distribution of binding poses in site A is relatively broad, showing a significant number of bound ligand modes featuring binding energies between −5.5 kcal/mol and −9 kcal/mol (Figure 8C). At the same time, there is a decay in the distribution as the number of ligand poses with binding scores below −9.0 kcal/mol is sharply decreased. A more detailed inspection of the distribution showed that binding modes of only certain molecules, particularly ZINC06524444 and ZINC08101192, can produce the lowest energy scores (Figure 8A,C). On the other hand, we also found that the energetic distribution of binding poses for site B featured two peaks at −5.0 kcal/mol and −8.0 kcal/mol, which indicated that binding to site B may also result in favorable binding (Figure 8B,D).

Of particular interest and significance is the identification of three top scoring compounds common to both sites: ZINC06524444, ZINC08101192, and ZINC08101193 (Figure 8A,B). These compounds show strong binding affinity and decreased variance at site B, while ZINC06524444 binds to site A with the lowest binding energy among top compounds and also demonstrates a favorable binding to site B, albeit with the most notable difference in variance between the sites (Figure 8B). These findings have interesting implications, showing that some drug-like molecules ability could target multiple allosteric pockets in the trimer with the favorable binding energy. The general availability of multiple druggable binding pockets in the S trimer that were found in our pocket detection analysis (Figure 1) suggests that only a fraction of high-affinity molecules could display selective preferences towards the experimentally validated allosteric sites A and B. In this context, it is particularly interesting that the top ranked compound ZINC6524444 displayed a clear binding preference towards the inter-protomer allosteric site A. By examining the structural and energetic aspects of binding to site A and identifying key residues targeted by ZINC6524444, we can establish hotspots of the allosteric site suggesting putative allosteric inhibitors. 

ZINC652444 is a close analog of flavonols that are known to be promising antiviral agents against SARS-CoV-2 infection [69,70]. It was observed that this class of molecules can block SARS-CoV-2 entry to ACE2 cells and have a high binding affinity to ACE2 and S protein, leading to the inhibition of S protein attachment to the ACE2 receptor [70,71]. Some studies reported the ability of flavonols to bind with the S protein and ACE2 receptor owing to the existence of ortho di-OH hydroxyl groups in the B ring of flavonols, leading to strong hydrogen bonds between the S protein and the flavonols [72,73,74]. This finding was confirmed by MD simulations and docking study of four flavonols, namely, fisetin, quercetin, isorhamnetin, and kaempferol, against the S protein, showing that these flavonol molecules may bind with the S protein by allosterically interfering with the ACE2 binding [75]. The antiviral entry activity of a panel of flavonoids including flavones, flavonols, and flavanols was evaluated using Biolayer interferometry (BLI) binding assay and molecular docking to predict the binding of selected bioactive flavonoids with the S-RBD protein [76]. By docking kaempferol, quercetin, and myricetin molecules with five binding pockets in the S-RBD, this study found that these molecules can bind to two sites where one of the suggested RBD pockets for binding is a potential allosteric binding site [76]. The results of our study supported these observations by expanding the docking screening to the full-length S trimer, showing that the flavonoid analogs have a potential to bind to distinct allosteric sites A and B that are important for the modulation of conformational changes and the stability of the closed S trimer.

Another group of molecules that emerged as top ranked compounds targeting both sites are ZINC08101192 and ZINC08101193 that are similar to tobramycin (ZINC8214692), which is an aminoglycoside antibiotic that has been implicated as an antiviral and believed to inhibit the S protein [77,78]. Tobramycin and related molecules were also identified among candidate molecules for the S-RBD binding by drug repositioning analysis of drug molecules contained in the Drug ReposER database and molecular docking [79]. Pharmacophore-based drug repurposing analysis of the DrugBank compounds using docking screening and MD simulations revealed that framycetin, kanamycin, and tobramycin were among the promising candidate compounds to inhibit S protein and viral entry [80]. 

**Figure 9:**
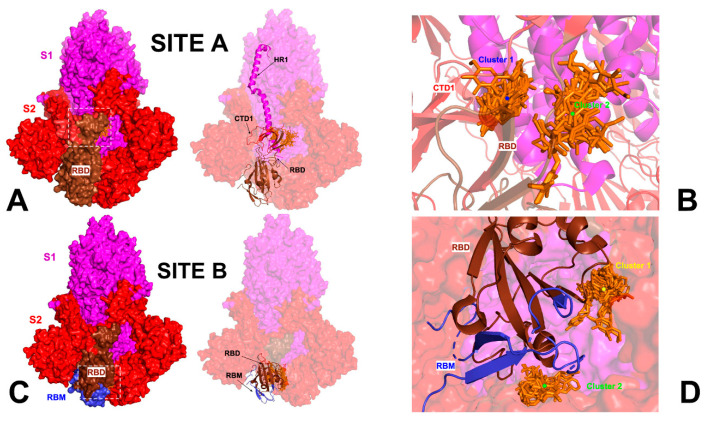
Structural analysis of the binding modes distribution for the top 10 lead compounds at site A and B. (**A**) The binding pocket at site A is situated between the S1 (red) and S2 (magenta) subunits. (**B**) A close-up of the two most common binding clusters in site A. (**C**) The binding pocket at site B. Bound ligands depicted in orange. (**D**) A close-up of the two most common binding clusters in site A. The S1 subunit is shown in red surface and S2 in magenta surface. Bound ligands are shown in orange sticks.

We proceeded with the structural analysis of the binding modes distribution for the top 10 lead compounds at each site revealing diversity of the ligand poses and the interactions between these compounds and the S protein. We analyzed common modes of binding at site A and site B. At site A, two clusters of binding were identified (Figure 9A,B). The most common binding pocket at site A is the least exposed, denoted cluster 1 (Figure 9A,B). Cluster 1 is tucked inside the S protein closer to HR1 (residues 910–985) and is covered by the SD1 (residues 528–591) and SD2 regions (residues 592–686). The binding mode for ligands forming cluster 1 may indicate its ability to interfere with the cooperative movements of SD1 and SD2. It is well recognized that allosteric coupling between the RBD movement and SD1–SD2 junction motions is a major force driving conformational changes in the S trimer [33]. Importantly, a detailed inspection of the key S residues making contacts in binding modes of cluster 1 (Supporting Information, Figure S1) revealed that F541, K557, T572, M740, D745, and L981 positions are located near hinge centers that are critical for global functional movements of the S trimer (Figure 3). Moreover, these positions were selected in the mutagenesis engineering studies to produce variants A570L, T572I, F855Y, and N856I of the S trimer that destabilized the closed state rather than stabilizing the open state [7]. Hence, the binding poses of cluster 1 suggest that high affinity molecules may insert themselves between the SD1 and SD2 of the S protein and thus have the potential to intervene with the conformational motion of the protein, maintaining the RBD in the “down” conformation. Our pocket analysis suggested that site A may be part of a larger site that is dynamically formed by S1 residues on one protomer (positions 541, 546, 547, 548, 549, 570, 572, 573, 587, 589, and 592) and S2 residues from the adjacent protomer (positions 1000, 740, 741, 744, 745, 855, 856, 966, 976, 977, and 978) (Figure 1B, pocket 4). The binding conformations of these compounds are positioned to potentially disrupt the movement of HR1 (residues 910–98) that interacts with HR2 during fusion. HR1 has been suggested as a potential target for fusion inhibitors, as the HR1 and HR2 regions of the S2 protein tend to be conserved [81]. In addition, a second cluster (cluster 2) was also found (Figure 9A,B) that primarily occupies the SD2 region and provides an additional subpocket for the binding of small molecules. While these two clusters represent the vast majority of binding poses, the local conformational changes in the SD1–SD2 region may lead to other ligand binding poses in this region that are less frequent.

At site B, we also found that the most common binding modes of the top 10 compounds can form two structural clusters that are situated between the S2 core and the S1 RBD and RBM (Figure 9C,D). The compounds tend to interact with the RBD residues R408, D405, R403, and Y505 that participate in shifting the RBD from the ‘down’ to ‘up’ state [35]. We also found that most of the binding poses (Figure 9D) mimic the experimentally verified binding of the sialic acid molecule [35] that interacts with the RBD residues R403, D405, R408, and Y505 of chain A, and the residues K378 and G404 of chain B (Supporting Information, Figure S1).

**Figure 10:**
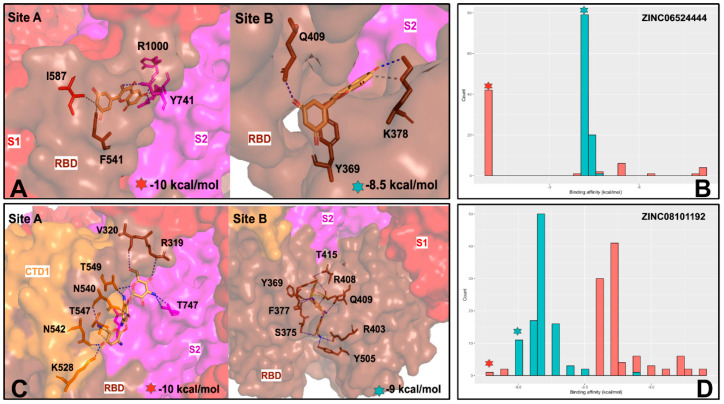
Structural analysis of the binding modes and binding energy distributions for ZINC06524444 and ZINC08101192 molecules, which emerged among the top 10 ranked compounds for both sites A and B. (**A**) The most common binding pose of ZINC06524444 (shown in sticks) at site A (shown in surface, S1 subunit in brown, S2 in magenta). (**B**) The distribution of binding energies for ZINC06524444 at site A (red bars) and site B (blue bars). (**C**) The most common binding pose of ZINC08101192 (shown in sticks) at site A (shown in surface, S1 subunit in brown, S2 in magenta). (**D**) The distribution of binding energies for ZINC08101192 at site A (red bars) and site B (blue bars). The S protein binding site residues making key interactions with ZINC06524444 and ZINC08101192 on panels A and C are annotated.

We now analyze in detail the structural and interaction signatures of binding revealed by ensemble-based docking for the top compounds targeting both sites, ZINC06524444 and ZINC08101192. ZINC06524444 showed clear energetic preferences towards allosteric site A by making a greater number of interactions with residues at Site A, including an additional hydrogen bond with residues on the S2 side of the interface, with D745 and Y741 of the α1/α2 helix region and R1000 of the central helix (CH) (Figure 10A). According to the predicted binding pose, this compound forms strong hydrophobic contacts with residues I587, F541, P589, and F855 that are located in the inter-domain hinge regions involved in modulation of S trimer transitions between the closed and open states. The distribution of binding poses and docking scores for ZINC06524444 showed a single dominant peak at site A (Figure 10B). The network of favorable contacts formed by ZINC06524444 with these residues leads to a stable binding pose with the S protein and can restrict global movements, thus enabling the ligand to interfere with the transitions to the open state and block binding with the ACE2. At the same time, ZINC06524444 binding is less energetically favorable due to fewer contacts that are mainly formed with Y369, K378, and Q409 residues (Figure 10A,B). Hence, our results indicated that a related hit molecule ZINC06524444 may target the inter-protomer S1–S2 allosteric site with high binding affinity. 

The second lead compound common to both sites is ZINC08101192, which is a close analog of the tobramycin drug. ZINC08101192 had the same mean binding affinity at site A and site B, with the distribution being marginally skewed towards higher binding affinity at site B (Figure 10 C,D). The binding mode at site A is stabilized by nine hydrogen bonds and favorable contacts with R319, V320, N540, T549, T547, N542, and K528 (Figure 10C). Interestingly, this molecule makes salt bridge with R319 and hydrophobic contacts with V320 residues which are major hinge centers that determine functional movements along slow modes (Figure 3). By targeting these key allosteric positions of the S trimer, ZINC08101192 molecule may directly interfere with the control of conformational changes and impede transitions between the closed and open states. As can be seen from the ensemble docking analysis, the distribution of binding energies pointed to the lowest score at site A (−10 kcal/mol) even though a notable peak of low binding energies was also found at site B (Figure 10D). At site B, ZINC08101192 forms salt bridges, with R408 implicated in the binding with ACE2 The favorable interactions at site B also include contacts with F377, S375, and Y369 residues (Figure 10D). Strikingly, these residues are the key RBD positions from the experimentally validated RBD allosteric pocket where the LA fatty acid binds [26,27]. The results of the docking screening revealed the structural and energetic preferences of ZINC08101192 to both allosteric sites by forming interactions with hinge residues at the S1–S2 junction while also showing a potential to target the experimentally known LA allosteric pocket and site B in the closed S trimer. 

In summary, the ensemble-based docking and binding analysis of library 1 of drug-like molecules revealed several high affinity compounds that can target both allosteric sites and establish a strong interaction network with the experimentally known allosteric hotspots of the S protein. Although the allosteric inhibition mechanisms for the proposed top compounds ZINC06524444 and ZINC08101192 are yet to be experimentally confirmed, our results probed structural and energetic determinants for targeting of experimentally validated allosteric pockets A and B. We found that targeting site A residues I587, F541, P589, and F855 that are located in the inter-domain hinge regions may elicit allosteric inhibition by interfering with the conformational equilibrium of the S trimer. The analysis of binding modes and interactions for the top compounds in site B showed that the key hotspots for allosteric targeting are F377, S375, Y369, R408, and Q409 residues that are also involved in the formation of the validated LA allosteric pocket. The important implication of these results is that molecules targeting this pocket through the formation of strong binding interactions with the identified allosteric centers may be a potential prerequisite for allosteric inhibition of the S protein.

### 2.4. Ensemble Docking and MM-GBSA Binding Free Energy Calculations of Allosteric Inhibitors 

In this section, we analyzed the results of ensemble-based docking and binding free energy analysis of series of allosteric modulators CPD1–CPD26 that were originally discovered in [33] and proposed to target allosteric site A. Unlike the initial study, we examined the structural and energetic aspects of binding for these allosteric inhibitors using conformational ensembles of the S protein and rigorous binding affinity computations. We focused our discussion on particularly revealing representative CPD molecules CPD7, CPD20, and CPD26 that, in the original study, demonstrated the strongest inhibitory effects in vitro and also revealed the strongest binding affinity constants in SPR assays [33]. 

**Figure 11:**
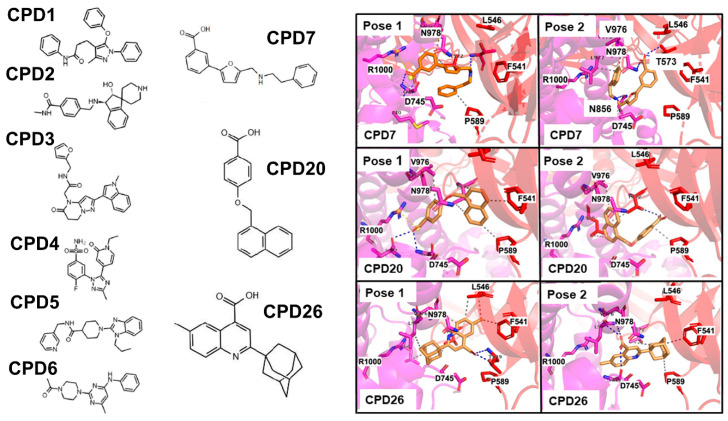
Structural analysis of binding poses for CPD compounds in the allosteric site A. (Left panel) The chemical formula for allosteric modulators CPD1–CPD7, CPD20, and CPD26 [33]. (Right upper panel) The most common binding poses 1 and 2 for CPD7 compound. (Right middle panel) The most common binding poses 1 and 2 for CPD20 compound. (Right lower panel) The most common binding poses 1 and 2 for CPD26 compound. The inhibitor is shown in sticks. The binding site residues involved in the interactions are shown in sticks and annotated.

**Figure 12:**
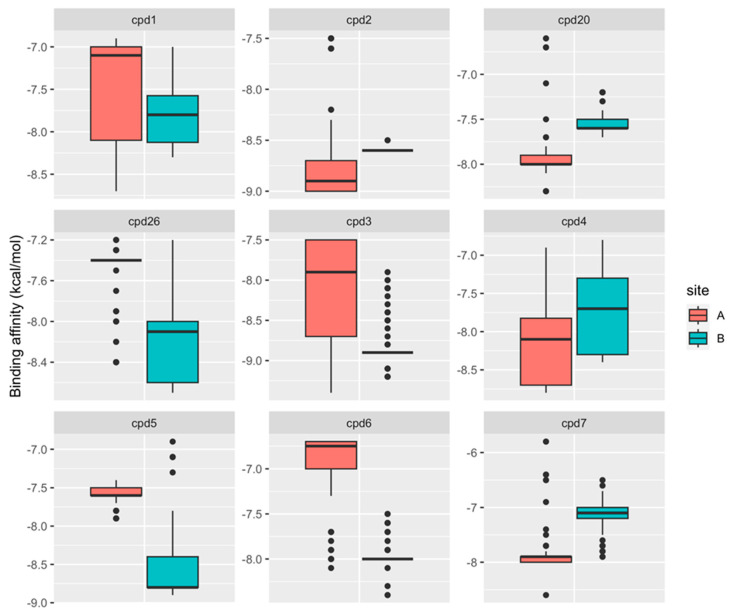
Binding affinity distributions obtained from ensemble docking of CPD1–CPD7, CPD20, and CP26 compounds at allosteric sites A and B. The box and whisker plot of binding affinity distributions for the CPD compounds at site A (in red) and site B (in blue) highlights the distribution of data points. The left and right sides of each box are the lower and upper quartiles. The box covers the interquartile interval, where 50% of the data are found. The vertical line that split the box in two is the median. The data points correspond to 200 docking scores obtained for each compound from 200 independent ensemble docking runs.

The ensemble-based docking and scoring analysis was performed for CPD compounds against both sites A and B. For simplicity and clarity of presentation, we focused primarily on discussing structural aspects and binding modes of CPD compounds in site A (Figure 11). We also present a complete analysis of the energetics of binding poses for CPD molecules against both sites (Figure 12). The analysis of favorable binding poses showed that the two most common binding conformations of CPD7 accounted for 75% of the total number of runs (Figure 11). In the first conformation, the benzene ring faces the S1 subunit, and the carbonyl group interacts with residues from the S2 subunit; however, in this confirmation, CPD7 forms a salt bridge instead of hydrogen bonds with R1000 (Figure 11, right upper panel). The structure is additionally stabilized on the S2 side by hydrogen bonding with M740 and G744. Instead of the nitrogen group bonding with D745, it forms a hydrogen bond with T573, which orients the benzene ring to hydrophobically interact with P589. In the second conformation, the benzene group faces the S2 subunit, and the carbonyl groups interact with both subunits, forming two hydrogen bonds with S1 residue T573 and one hydrogen bond with S2 residue N978 (Figure 11, right upper panel ). The nitrogen group hydrogen bonds with Asn856 of the S2 subunit and the benzene ring hydrophobically interacts with S2 residues Leu977 and Val976. These two major conformations of CPD7 displayed similar occupation frequency. The distribution of binding energies showed a preference for binding pose 2, in which CPD7 makes important contacts with hinge positions F541 and P589. 

The two most common bound conformations for the CPD20 molecule account for 86% of the total distribution, with the binding pose 2 occurring more frequently (pose 1–20%, pose2–66%) (Figure 1, right middle panel). The binding pose 1 of CPD20 involved a salt bridge with R1000, as well as interactions with I742 and G744 of the S2 subunit. The aromatic rings were primarily stabilized through hydrophobic interactions with S1 residues F541, P589, and T572 (Figure 11, right middle panel). In the second more common binding pose, the aromatic rings face the S2 subunit, making hydrogen bond with T572 and hydrophobic interactions with S2 residues P589, L966, and V976. The ensemble docking indicated that the most frequent and energetically favorable binding mode (pose 2 for both CPD7 and CPD20) is quite similar, in which the ligands are engaged in a network of contacts with S2 positions implicated in functionally important hinge sites which may explain their allosteric inhibition effect. Our results suggest that using this preferable mode of binding CPD7 and CPD20 molecules may target hinge sites and allosterically interfere with the conformational equilibrium preventing S protein from stabilization of the open form and consequently inhibiting the ACE2 binding. Interesting results were obtained for the most active and strongest binder CPD26 [33] for which we observed the binding pose 1 as the most dominant (~ 50% occupancy) (Figure 11, right lower panel). In this bound conformation, CPD26 makes two hydrogen bonds with S2 residue T549 and hydrophobic interactions on both the S1 and S2 subunits. At the same time, the flipped binding mode 2 can leverage strong hydrophobic contacts with hinge position P589, F541 (Figure 11, right lower panel). While the energetically similar but structurally flipped binding modes of CPD26 may indicate certain inherent deficiencies of the docking scoring function, it is also possible that this allosteric inhibitor may employ several alternative but functionally viable positions in the S1–S2 site to maximize the binding interactions and enhance the binding affinity. 

The energetics of multiple bound orientations of CPD26 is reflected in the distribution of binding scores (Figure 12). The binding affinity distributions using ensemble docking to multiple S protein conformations showed an appreciable dispersion owing to both multiple binding modes of CPD compounds and conformational plasticity of the inter-protomer allosteric site (Figure 12). We found that on average, the binding scores for ligands poses were more favorable in site A. In particular, CPD2, CPD4, CPD7, and CPD20 displayed a clear energetic preference towards the allosteric site A (Figure 12). Notably, the experimentally best binder CPD26 [33] showed an appreciable binding affinity and broad distribution of favorable binding scores in both sites A and B as compared to other moderate binders such as CPD7 and CPD20 (Figure 12). This suggested that the observed conformational variability of the energetically favorable binding poses for CPD26 may contribute to the experimentally observed stronger binding affinity and inhibitory activity [33]. 

Notably, binding energies of ligand poses in molecular docking serve as approximate evaluations of binding affinities. To enable a more physically rigorous evaluation of binding free energies for the CPD compounds and validate the predicted binding modes of the inhibitors, we used conformational equilibrium ensembles obtained from simulation and the molecular mechanics/generalized Born surface area (MM/GBSA) method [82] for rescoring of predicted docking poses. The MM-GBSA approach presents a robust method that combines computational efficiency and prediction accuracy [82]. While accurate MM-GBSA evaluations of absolute binding free energies may be prone to errors associated with summing up large contributing terms, the application of the MM-GBSA methodology is particularly suitable for the assessment of relative binding energy differences. The virtual screening pipeline has been extended by ensemble-average MM/GBSA rescoring. The central objective of this computational analysis is to compare predicted MM-GBSA estimates of binding free energies with the experimental binding affinities identified for CPD compounds using SPR experiments and anti-SARS-CoV-2 assays [33]. These compounds demonstrated experimental ability to interact with the S protein and yield the micromolar binding affinities (Table 1). We compared our predictions with the experimental binding constants from the original study [33]. 

**Figure 13:**
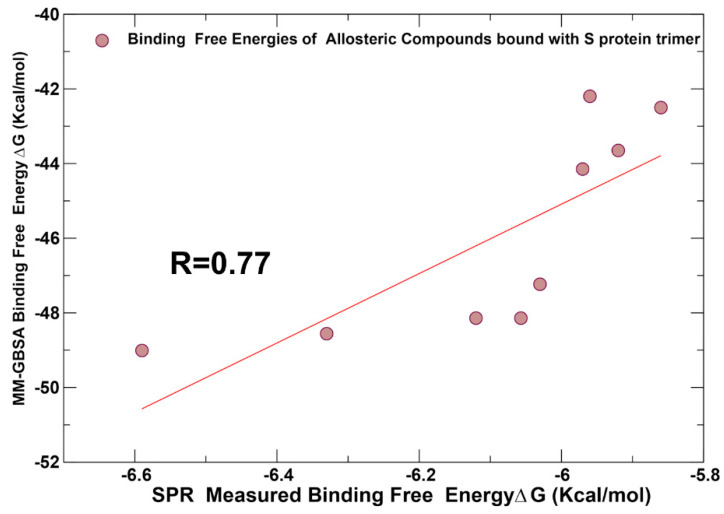
The scatter plot between the MM-GBSA computed binding energies and the experimentally measured in SPR assays binding affinities [33] for nine allosteric compounds: CPD1, CPD2, CPD3, CPD4, CPD5, CPD6, CPD7, CPD20, and CPD26. The Peason correlation coefficient is R = 0.77.

The results of the MM-GBSA computations showed good agreement with the experimental binding constants [33] (Figure 13) with the Peason correlation coefficient R = 0.77 suggesting that ensemble docking screening and MM-GBSA postprocessing analysis provide a reasonable approach for predicting binding affinities of these allosteric inhibitors. The moderate binding of these compounds detected experimentally may also reflect the observed heterogeneity of the binding poses and adaptability of the allosteric sites. Our computational predictions of MM-GBSA binding affinities demonstrated that the best binding affinities are produced by the CPD7, CPD20, and particularly CPD26 compounds (Table 1, Figure 13). These results agree with the experimental studies showing that the binding affinities obtained from SPR experiments identified these three compounds as the best binders [33]. Moreover, the best compounds, CPD7, CPD20, and CPD26, were proven to be able to inhibit SARS-CoV-2 at a concentration of 100 μM and showed inhibitory effects by intervening with ACE2 binding in the experimental assays [33]. This suggests that these CPD compounds may inhibit viral entry and ACE2 binding through binding to the identified allosteric site and blocking conformational transitions. This rationale is supported by our analysis showing that the allosteric site located at the junction of SD1 and SD2 regions is formed by hinge residues of the inter-domain options that function as regulatory switch centers governing the population shifts between closed and open forms. 

To summarize, we examined a library of allosteric molecules, CPD1–CPD26 [33], for binding to allosteric sites, and computed binding affinities of these molecules based on the predicted bound structures and generated conformational ensembles. Using these computational approaches, we also identified the key interactions and allosteric hotspot residues that are responsible for effective ligand binding in allosteric sites. The results of this study suggest that combining conformational dynamics analysis, binding pocket detection, and ensemble-based ligand docking with multiple conformational states of the S protein trimers enables robust characterization and predictions of putative allosteric modulators that can target experimentally validated allosteric binding sites. The ensemble-based docking strategies [83] have demonstrated a significant promise and robust performance in large scale virtual screenings and detecting high-affinity binding conformations when using conformational protein ensembles rather than static structures. 

This study also presented evidence of significant challenges in the identification of allosteric modulators of the S protein and validation of the allosteric inhibitory mechanisms. The presence of multiple cryptic pockets in the S trimer that appear not only in the flexible S1 subunit but could be also found in more rigid S2 regions point to the diversity of potential allosteric sites and difficulties in the prediction of functional binding modes for allosteric modulators. The results of our study revealed several conserved and druggable cryptic pockets in the regulatory hinge regions of CTD1-S2 and S2 that engage in communication between functional elements of S2 and RBD. The targeting of allosteric pockets away from the binding interface with ACE2 could also give rise to distinct allosteric inhibitory mechanisms that remain effective even when the S protein is bound to ACE2 and lead to global destabilization or weakening of the inter-protomer interactions and thus compromising the viral entry. Recent HDX-MS experiments showed that ACE2 binding enhances dynamics at a distal S1/S2 cleavage site while restricting dynamics of the stalk hinge of the S2 subunit [23]. As a result, allosteric targeting of the stalk and proteolysis sites of the S protein bound to ACE2 may compromise long-range couplings and the ability of the S2 subunit to promote host–viral membrane fusion and viral entry. HDX-MS and structural studies discovered a cryptic hinge epitope located in the S2 region between the CH and HR1 segments suggesting that targeting this pocket may lead to allosteric inhibition the S conformational changes required for fusion of the viral envelope and target cell membrane [46]. These investigations underscored a considerable diversity of potential allosteric pockets that can elicit allosteric inhibition in alter binding with the ACE2 receptor via long-range conformational changes [84]. Recent analysis of antibody-mediated neutralization of S protein and its variants showed that weak neutralizers can bind to RBD-up position in the presence of ACE2, which leads to allosteric relay across the S1 and S2 subunits and result in the increased conformational dynamics. These antibodies mediate neutralization via destabilization of the S trimer, while some other neutralizers can allosterically induce decreased dynamics [85]. In both cases, allosteric inhibition mechanisms could remain effective in the presence of ACE2 and compromise S activity via modulation of dynamic effects. It is also believed that SARS-CoV-2 may lose the ability to infect new host cells due to allosterically impaired interaction between the ACE2 receptor and the viral RBD. The identification of allosteric sites on the ACE2 receptor suggested that altering the biophysical properties of the ACE2 receptor by modulating an allosteric site of ACE2 may enable allosteric inhibition of the S–ACE2 interactions [86].

Another important and challenging area of analysis is the potential off-target effects of the identified allosteric inhibitors. In a more general context, many agents and small molecules that show inhibition of SARS-CoV-2 replication could exploit off-target effects by interfering with viral replication through non-specific effects [87]. It was recently found that Imatinib inhibits SARS-CoV-2 infection via an off-target effect where rather than impairing S–ACE2 interactions the drug can stabilize the S protein via allosteric binding [88]. Our analysis showed the importance allosteric inhibitor selectivity and promiscuity where some allosteric molecules are more selective against a particular allosteric pocket, while some other validated allosteric molecules display favorable binding energetics with multiple allosteric pockets. Given a significant number of cryptic binding pockets on the S surface, it is important to identify and validate targeted allosteric pockets which can facilitate understanding of the inhibitory mechanisms. In this context, the experimental validation of the LA allosteric pocket as a primary target for many active compounds binding to this site with high affinity and stabilizing the inactive conformation of the S protein exemplifies targeted allosteric intervention [64]. Our study indicated that allosteric molecules may target the conserved inter-protomer allosteric pocket (site A) and RBD–RBD pocket (site B) that includes residues from the LA binding site. We suggest that conservation sequence and structure conservation of these pockets in highly pathogenic human coronaviruses may be exploited for the validation and development of allosteric antiviral agents, with broad-spectrum antiviral activities. 

## 3. Materials and Methods

### 3.1. Coarse-Grained Brownian Dynamics Simulations

All structures were obtained from the Protein Data Bank [89]. During structure preparation stage, protein residues in the crystal structures were inspected for missing residues and protons. Hydrogen atoms and missing residues were initially added and assigned according to the WHATIF program web interface [90]. The missing loops in the studied cryo-EM structures of the SARS-CoV-2 S protein were reconstructed and optimized using template-based loop prediction approach (ArchPRED) [91]. The side chain rotamers were refined and optimized through the SCWRL4 tool [92]. The protein structures were then optimized using atomic-level energy minimization with composite physics and knowledge-based force fields as implemented in the 3Drefine method [93]. Coarse-grained Brownian dynamics (BD) simulations were conducted using the ProPHet (probing protein heterogeneity) approach and program [94,95,96,97]. BD simulations were based on a high resolution CG protein representation [98] of the SARS-CoV-2 S Omicron trimer structures that can distinguish different residues. In this model, each amino acid is represented by one pseudo-atom at the Cα position, and two pseudo-atoms for large residues. The interactions between the pseudo-atoms were treated according to the standard elastic network model (ENM) in which the pseudo-atoms within the cut-off parameter, *R*_c_ = 9 Å are joined by Gaussian springs with the identical spring constants of *γ* = 0.42 N m^−1^ (0.6 kcal mol^−1^ Å^−2^. The simulations used an implicit solvent representation via the diffusion and random displacement terms and hydrodynamic interactions through the diffusion tensor using the Ermak–McCammon equation of motions and hydrodynamic interactions, as described in the original pioneering studies that introduced Brownian dynamics for simulations of proteins [99]. The stability of the SARS-CoV-2 S Omicron trimers was monitored in multiple simulations with different time steps and running times. We adopted Δ*t* = 5 fs as a time step for simulations and performed 100 independent BD simulations for each system using 100,000 BD steps at a temperature of 300 K. The CG-BD conformational ensembles were also subjected to all-atom reconstruction using PULCHRA method [100] and CG2AA tool [101] to produce atomistic models of simulation trajectories.

### 3.2. Machine Learning-Based Discovery of Cryptic Pockets and Network-Based Ranking of Allosteric Pocket Propensities and Allosteric Binding Sites

We used two different complementary approaches for enumerating the putative binding pockets in the conformational ensembles of the S-BA1 and S-BA.2 structures. A template-free P2Rank approach is among the most efficient and fast available ML tools for the prediction of ligand binding sites, combining sequence and structural data to rank potential binding sites based on their likelihood of binding a specific ligand [60]. P2Rank uses support vector machine (SVM), random forests (RF), and artificial neural networks (ANNs) to learn the ligand ability of a local chemical environment that is centered on points placed on the protein’s solvent-accessible surface [60]. We also used the prediction of allosteric sites server (PASSer) approach [61,62] and the PASSer learning to rank (LTR) model that is capable of ranking pockets in order of their likelihood to be allosteric sites [63]. Using P2Rank and PASSer LTR approaches, we identified binding pockets in the conformational ensembles and computed P2Rank-predicted residue pocket probability. The reported top binding pockets for each protein structure correspond to consensus P2Rank/LTR predicted sites. 

### 3.3. Virtual Screening Protocol Using Autodock Vina 

A typical virtual screening pipeline begins with the ligand molecules, which can be downloaded from public databases such as PubChem or the ZINC database (Supporting Information, Figure S2). These molecules are structure-data files (sdf) that encode chemical structure properties. These files are then converted to protein data bank (PDB) format and PDBQT format, which includes additional information about atom charges and types that is necessary to simulate the most accurate interactions between the ligand and receptor. These conversions are performed using Open Babel software. For virtual screening pipeline the structure of the close S trimer structures (pdb id 6VXX, 6X2C) were used. AutoDock Vina [65] was used for the prediction of ligand binding modes. AutoDockTools [102] was used for reading PDB files, adding H’s and Gasteiger charges, and generating AutoDock input (pdbqt) files. AutoGrid with a grid spacing of 0.375 Å (AutoDock: spacing parameter) was used as a default spacing parameter. To perform virtual screening, the pdbqt files of all ligands and the receptor are submitted as input to Vina, along with a configuration file. The configuration file specifies the exact coordinates of the docking site as a grid box. The configuration file references a script that specifies the number of docking runs per ligand (Supplemental Information). Site A is located at the S1–S2 (Chain B) junction, with grid parameters (Å) x = 40 y = 40 z = 40 and Center (Å) x = 225.178, y = 174.656, z = 213.746. Site B is located between Chain B and Chain C, with grid parameters (Å) x = 40 y = 40 z = 40 and Center (Å) x = 196.983, y = 213.878, z = 256.849. All ligand-receptor conformations will be confined to this search space. The output of Vina includes a text file summarizing the top nine ligand-receptor conformations and their corresponding binding affinities. Vina also outputs each conformation as a pdbqt file for visualization and analysis. VINA scoring function is based on an X-score, which is a consensus scoring (CS) function based on three distinct strategies to represent the favorable contribution of the desolvation event related to the hydrophobic effect: hydrophobic surface (X-Score^HS^), hydrophobic matching (X-Score^HM^), and hydrophobic contact algorithms (X-Score^HC^) [103,104]. Unlike AutoDock, Vina does not use explicit hydrogen atoms or the partial charges for atoms in the simulations. The binding of different compounds was characterized in terms of the predicted binding affinities for the different binding modes of each compound in each cluster, as well as the relative population distribution of these compounds in each cluster. The predicted binding affinities for each binding mode were obtained from the AutoDock Vina output. 

### 3.4. MM-GBSA Binding Free Energy Computations

The binding free energies were initially computed to use the MM-GBSA approach [82,105,106,107]. The binding free energy for each S protein complex was obtained using the following formulas:

(1) $$\Delta G_{\mathrm{bind}}=G_{S-\mathrm{ligand}}-G_{S}-G_{\mathrm{ligand}}$$

(2) $$\Delta G_{bind,MMGBSA}=\Delta E_{MM}+\Delta G_{sol}-T\Delta S$$

where Δ*E_MM_* is total gas phase energy (sum of Δ*E_internal_*, Δ*E_electrostatic_*, and Δ*Evdw*), and Δ*Gsol* is the sum of polar (Δ*G_GB_*) and non-polar (Δ*G_SA_*) contributions to solvation. The binding free energy with MM-GBSA was computed by averaging the results of computations over 1000 samples from the equilibrium ensembles. A common strategy to reduce noise and cancel errors in MM-GBSA computations is to run MD simulations on the complex only, with snapshots taken from a single trajectory to calculate each free energy component. The binding energies between the docked ligands was calculated for equilibrium samples with thermal_mmgbsa.py python script provided by Schrödinger which takes the trajectory file, splits it into individual snapshots, runs the Prime-MMGBSA calculations on each frame, and yields the average calculated binding energy.

## 4. Conclusions

In the current study, we employed an integrative approach and a pipeline which included (a) detection and characterization of allosteric pockets in the S-RBD and S trimer proteins, (b) CG-BD simulations with atomistic reconstruction of equilibrium trajectories for the S protein timers, (c) ensemble-based ligand docking using AutoDock Vina tools against conformational ensembles multiple protein states of the S closed trimers, and (d) binding free energy evaluation for allosteric modulators. Using ensemble docking screening of small molecules against several allosteric binding pockets of the S protein, we examined structural and binding preferences of ligand libraries against major allosteric binding sites in the full-length S protein trimer that were experimentally validated as targets of allosteric inhibitors. The results demonstrated a good agreement between computational and experimental binding affinities, providing support to the predicted binding modes and suggesting key interactions formed by the allosteric ligands to elicit the experimentally observed inhibition. We characterized structural and energetic determinants of allosteric binding for a series of experimentally known allosteric molecules, indicating a potential mechanism of allosteric modulation by targeting the hinges of the inter-protomer movements and blocking conformational changes between the closed and open spike trimer forms. Further molecular modifications are required to transform these compounds into potential therapeutic agents because the binding affinities of CPD compounds are relatively moderate and in the micromolar range. It can be expected that high-affinity modulators binding to this site with stronger binding affinities would produce a more selective binding mode; more specificity is needed for viable therapeutic agents. 

This study may have implications for understanding the effects of protein heterogeneity and ensemble-based view of ligand binding, providing useful insights into mechanisms of the allosteric inhibition of S proteins. Given the potential to significantly affect the virus life cycle by binding to the explored inter-domain allosteric site, a comprehensive understanding of the structural and energetic determinants of targeting these pockets and an identification of allosteric residue hotspots may aid in the development of novel, potentially broad-spectrum therapeutic agents. Our findings can be also important for understanding dynamics and hotspots on druggable allosteric pockets that can be used for both site-specific and allostery-inspired therapeutic intervention strategy targeting distinct conformational states of the SARS-CoV-2 Omicron variants. This may enable engineering of allosteric modulators that could rationally target a complex landscape of the S proteins.

## Figures and Tables

**Table 1:** MM-GBSA binding energies for the CPD compounds *.

System	MM-GBSA Computed Δ*G*_bind (kcal/mol)_	Experimental Binding Affinity Δ*G*_bind (kcal/mol)_	Experimental Binding Constant from SPR Measurements [33] KD (μM)
CPD1	−48.14	−6.057	36.2 μM
CPD2	−42.51	−5.86	50.4 μM
CPD3	−42.22	−5.96	42.3 μM
CPD4	−47.23	−6.03	37.7 μM
CPD5	−43.65	−5.92	44.9 μM
CPD6	−44.15	−5.97	41.3 μM
CPD7	−48.14	−6.12	32.0 μM
CPD20	−48.56	−6.33	22.6 μM
CPD26	−49.01	−6.59	14.6 μM

* Allosteric modulators and their binding affinities obtained from SPR experiments [33].

## Data Availability

Data are fully contained within the article. Crystal structures were obtained and downloaded from the Protein Data Bank (http://www.rcsb.org (accessed on 20 February 2024)). The rendering of protein structures was completed with interactive visualization program UCSF ChimeraX package (https://www.rbvi.ucsf.edu/chimerax/, accessed on 25 February 2024) and PyMOL (https://pymol.org/2/, accessed on 16 February 2024).

## Supplementary Materials

The following supporting information can be downloaded at https://www.mdpi.com/article/10.3390/ijms25094955/s1.
