# Supplementary material for: Exploring Binding Pockets in the Conformational States of the SARS-CoV-2 Spike Trimers for the Screening of Allosteric Inhibitors Using Molecular Simulations and Ensemble-Based Ligand Docking
Source: Int J Mol Sci. 2024 May 1;25(9):4955. doi: 10.3390/ijms25094955 (PMC11084335; doi:10.3390/ijms25094955)
Supplement: Supplementary file 1 [file ijms-25-04955-s001.zip › SUPPLEMENTARY_MATERIALS_REVISED.pdf]

Supplementary Materials

Exploring Binding Pockets in the Conformational States of the SARS-CoV-2 Spike Trimers for Screening of Allosteric Inhibitors Using Molecular Simulations and Ensemble-Based Ligand Docking

Grace Gupta,[1] Gennady Verkhivker[1,2]*

Keck Center for Science and Engineering, Graduate Program in Computational and Data Sciences, Schmid College of Science and Technology, Chapman University, Orange, CA 92866, USA; grgupta@chapman.edu

[2] Department of Biomedical and Pharmaceutical Sciences, Chapman University School of Pharmacy, Irvine, CA 92618, USA

* Correspondence: verkhivk@chapman.edu; Tel.: +1-714-516-4586

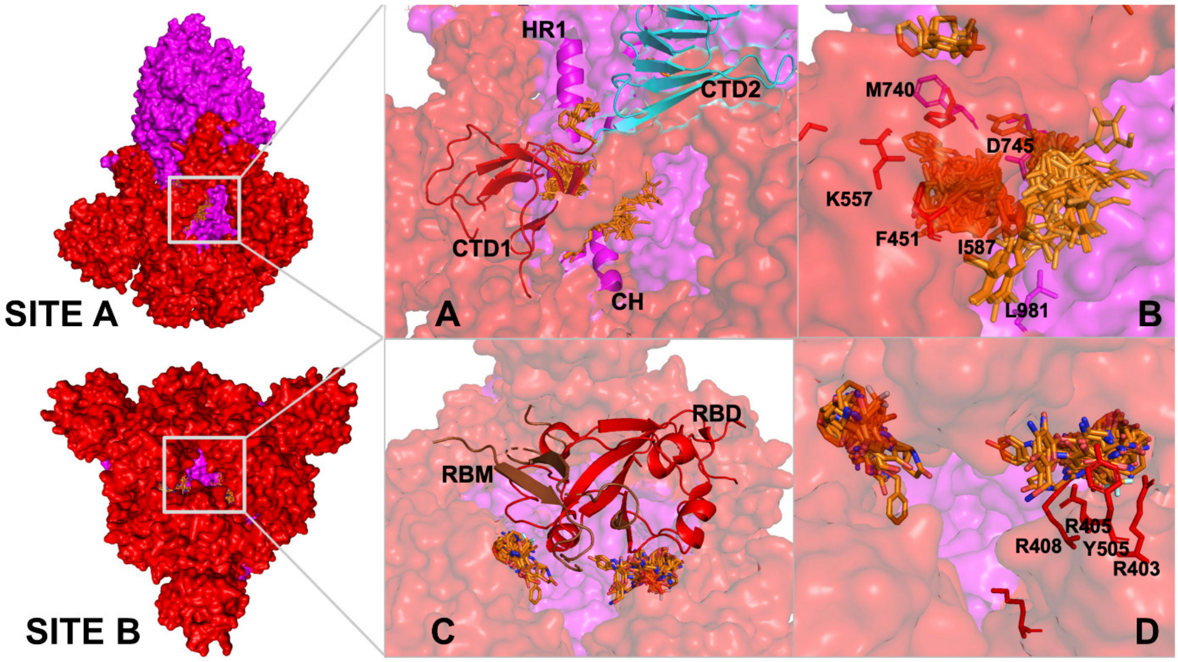

Figure S1. Structural analysis and closeups of the binding pockets and clusters of ligand poses (in default stick colors) of top compounds at site A and B. (A) The binding pocket at Site A is situated between the S1 (red) and S2 (magenta) subunits. (B) A close-up of the two most common binding clusters in site A. Bound ligands are depicted in orange sticks. (C) The binding pocket at Site B and two major clusters of binding poses. The bound ligands are shown in default colored sticks. (D) A close-up of the two most common binding clusters in site A. The S1 subunit is shown in red surface and S2 in magenta surface. The bound ligands are shown in default colored sticks. The binding site residues that are shown are in red sticks and annotated with residue name/number in bold black color.

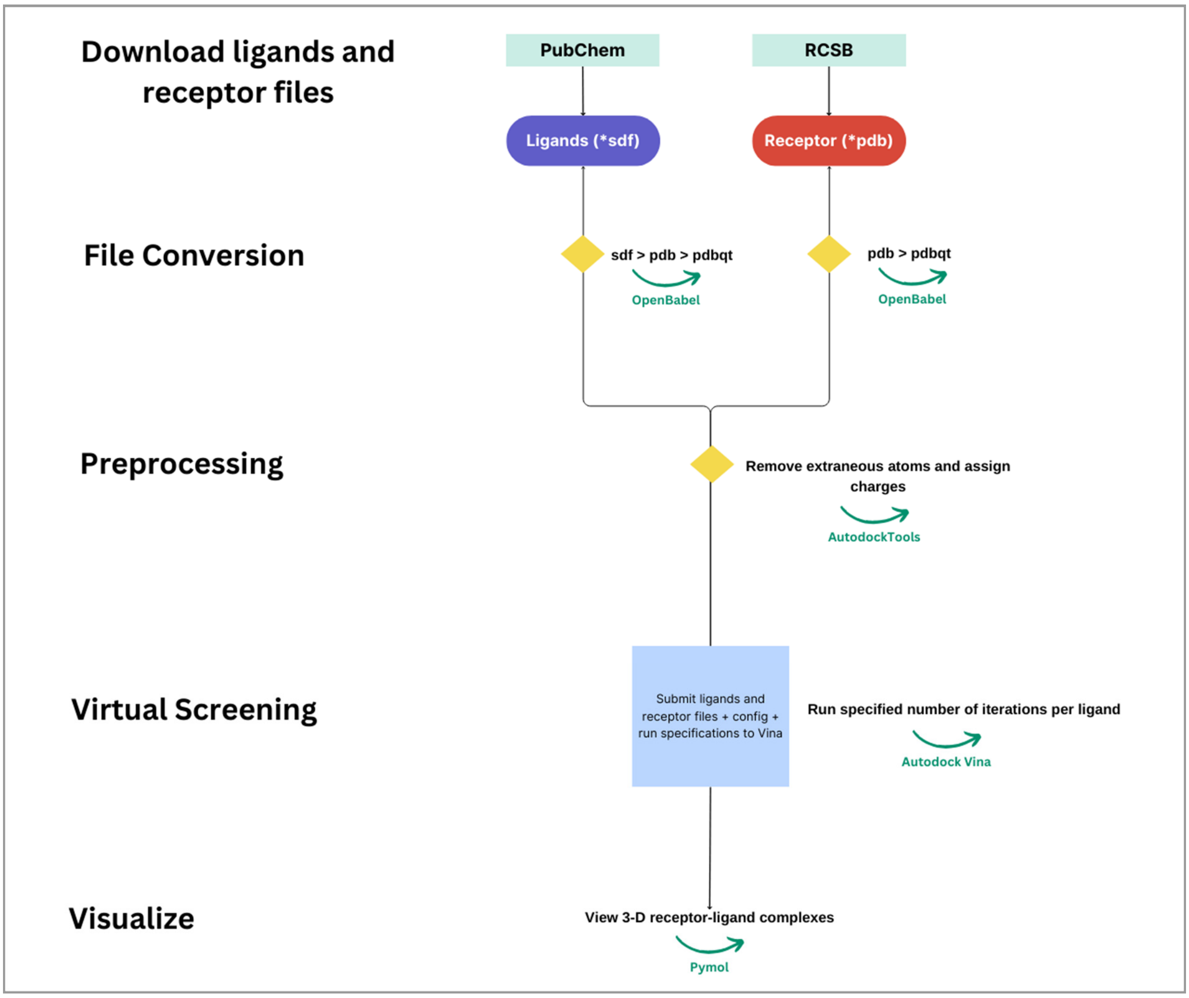

Figure S2. A schematic flow-chart of the virtual screening protocol used in this study.

Auto dock Scripts

A.1 Iteration Script

```perl
#!/usr/bin/perl
print"Ligand_file:\t";
$ligfile=<STDIN>;
chomp $ligfile;
open (FH,$ligfile)||die "Cannot open file\n";
@arr_file=<FH>;

for($i=0;$i<@arr_file;$i++)
{
print"@arr_file[$i]\n";
@name=(split(/\./,@arr_file[$i]))[0];
}
for($i=0;$i<@arr_file;$i++)
{
        chomp @arr_file[$i];
        print"@arr_file[$i]\n";
        mkdir @name;
        for($j=1; j<=3; $j++)
        {
                system("vina --config conf.txt --ligand @arr_file[$i] --log @name/@arr_file[$i]_[$j]_log.log");
        }
}
```

A.2 Output Script

```bash
#!/bin/bash

echo 'num, zincid, filename,mode,affinity,dist_rmsd,best_mode_rmsd' | tee -a output.csv

ligfile="$1"
while read -r line; do
        name=$(echo "$line" | cut -f 1 -d '.')
        echo "$name"
        for i in $name/*.log; do
                echo -n $name, | tee -a output.csv
                id=$(grep -A1 "<zinc_id>" $name.sdf | tail -n 1)
                echo -n $id, | tee -a output.csv
                echo -n $i | tee -a output.csv
                result=$(grep -A3 'mode' $i | tail -n 1 | tr -s " " | sed 's/ /, /g')
                if [ -z "$result" ] ; then echo 'NA,NA,NA,NA'| tee -a output.csv; else echo $result | tee -a output.csv; fi
        done
done < "$ligfile"
```

A.3 Cluster Scripts

```bash
#!/bin/bash

ligfile="$1"
while read -r line; do
        name=$(echo "$line" | cut -f 1 -d '.')
        echo "$name"
        cd $name
        for ((i=79; i<101; i++)); do
                echo "$i"
                vina --config ../conf_6m0j_fullgrid.txt --ligand $line --out [$name]_[$i]_out.pdbqt --log [$name]_[$i]_log.log
        done
        cd ..
done < "$ligfile"
```

```
#!/bin/bash

jobid="$1"

cat > job_$1.sbatch << "END"

#SBATCH --time=16-0
#SBATCH --ntasks-per-node=1
#SBATCH --cpus-per-task=1
#SBATCH --mem-per-cpu=40G
#SBATCH --partition=cpu-long.q
#SBATCH --output=slurm_$1.out
#SBATCH --job-name=serialvina_$1
#SBATCH --mail-type=END,FAIL
#SBATCH --mail-user=grgupta@chapman.edu

module load anaconda3/current
source /cm/shared/apps/anaconda3/etc/profile.d/conda.sh
conda activate myprojenv

(time ./vina_iterate.sh $1.txt) &> $1_time.txt

END
```

```
#!/usr/bin/env bash

#SBATCH --time=16-0
#SBATCH --ntasks-per-node=1
#SBATCH --cpus-per-task=1
#SBATCH --mem-per-cpu=40G
#SBATCH --partition=cpu-long.q
#SBATCH --output=slurm_zinc_1.out
#SBATCH --job-name=zincvina_x1
#SBATCH --mail-type=END,FAIL
#SBATCH --mail-user=grgupta@chapman.edu

module load anaconda3/current
source /cm/shared/apps/anaconda3/etc/profile.d/conda.sh
conda activate myprojenv

(time ./vina_iterate.sh zinc_1.txt) &> zinc_1_time.txt
```
