# Supplementary material for: Exploring Binding Pockets in the Conformational States of the SARS-CoV-2 Spike Trimers for the Screening of Allosteric Inhibitors Using Molecular Simulations and Ensemble-Based Ligand Docking
Source: Int J Mol Sci. 2024 May 1;25(9):4955. doi: 10.3390/ijms25094955 (PMC11084335; doi:10.3390/ijms25094955)

Supporting Information

Exploring Binding Pockets in the Conformational States of the SARS-CoV-2 Spike Trimers for Screening of Allosteric Inhibitors Using Molecular Simulations and Ensemble-Based Ligand Docking

Grace Gupta,^1^ Gennady Verkhivker^1,2^*

Keck Center for Science and Engineering, Graduate Program in Computational and Data Sciences, Schmid College of Science and Technology, Chapman University, Orange, CA 92866, United States of America

^2^ Department of Biomedical and Pharmaceutical Sciences, Chapman University School of

Pharmacy, Irvine, CA 92618, United States of America

***** Correspondence: verkhivk@chapman.edu; Tel.: +1-714-516-4586 (G.V)

Received: date; Accepted: date; Published: date

A.1 Iteration Script


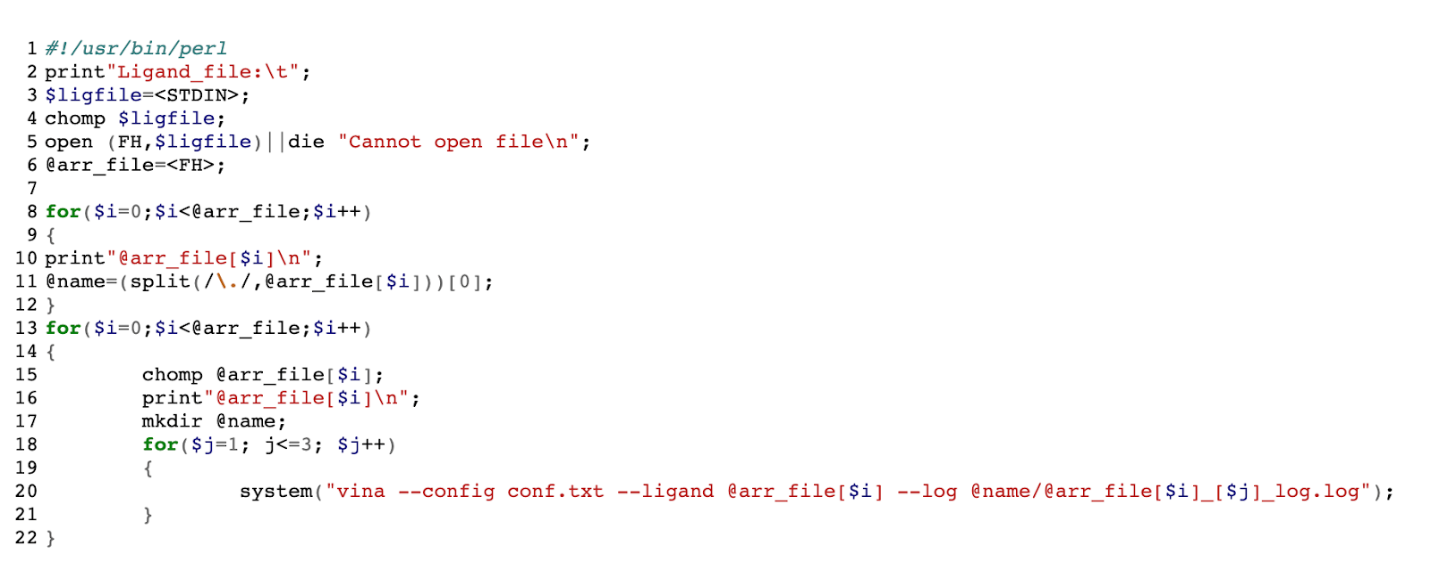


A.2 Output Script


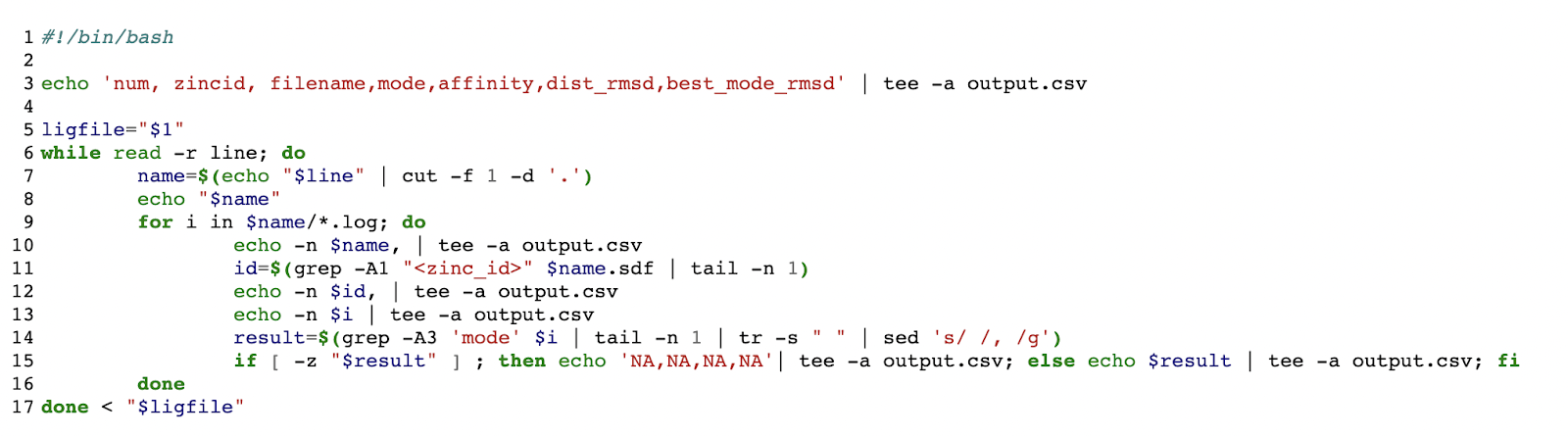


A.3 Cluster Scripts


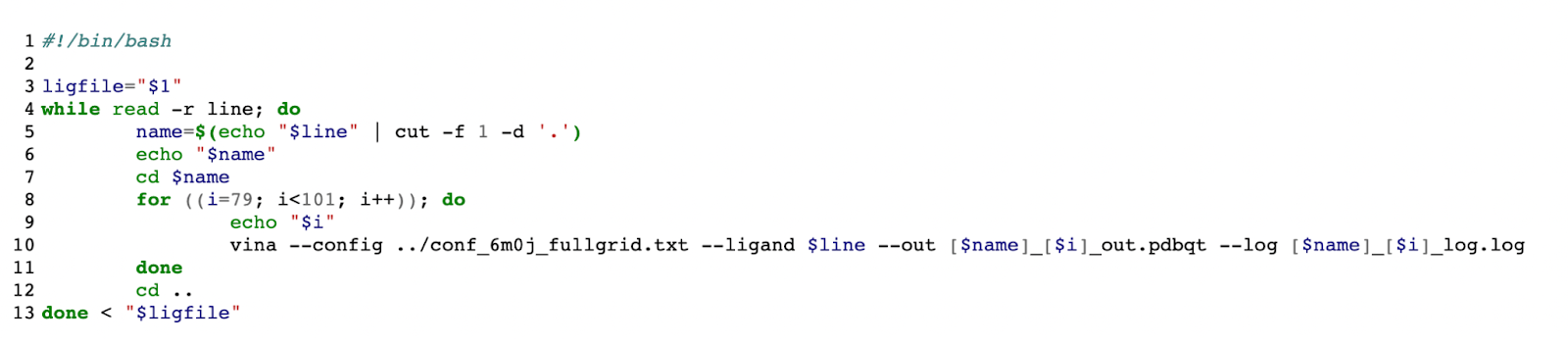


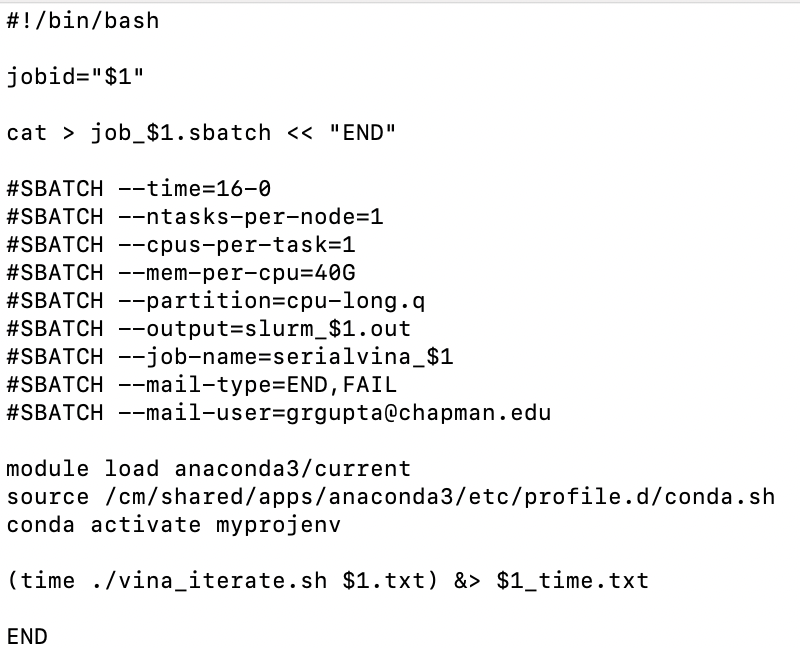


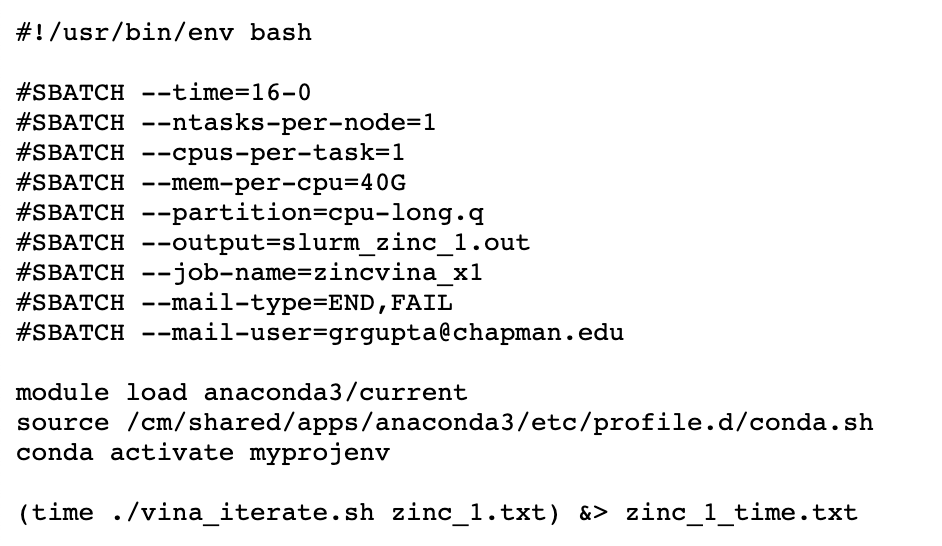

Supplement: Supplementary file 1 [file ijms-25-04955-s001.zip › Suppporting_Information_scripts/Supporting Information.docx]
