# Supplementary figures and images for: Exploring Binding Pockets in the Conformational States of the SARS-CoV-2 Spike Trimers for the Screening of Allosteric Inhibitors Using Molecular Simulations and Ensemble-Based Ligand Docking
Source: Int J Mol Sci. 2024 May 1;25(9):4955. doi: 10.3390/ijms25094955 (PMC11084335; doi:10.3390/ijms25094955)

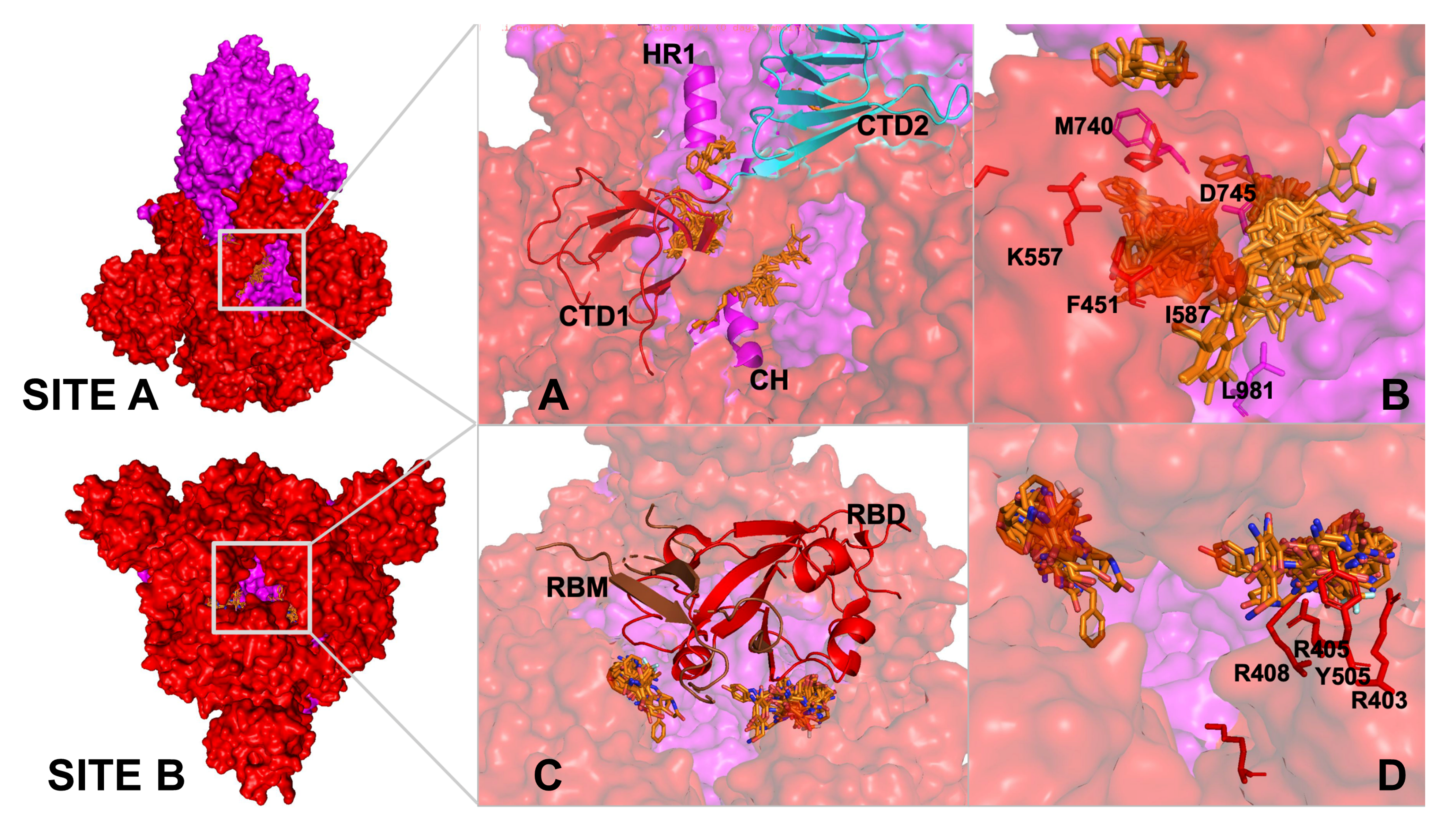

Supplement: Supplementary file 1 [file ijms-25-04955-s001.zip › FigureS1.tif]

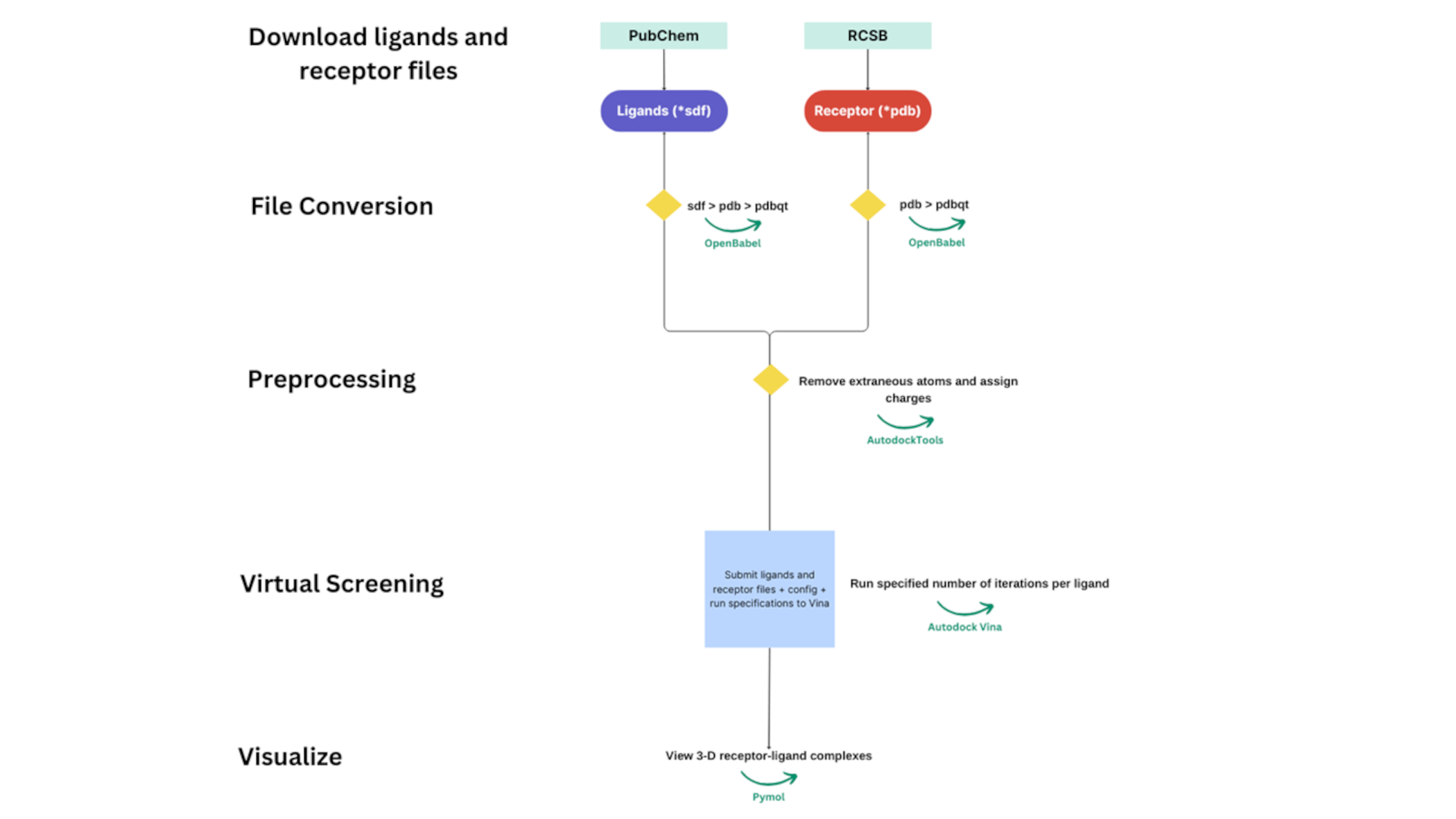

Supplement: Supplementary file 1 [file ijms-25-04955-s001.zip › FigureS2.tif]
